# Ochre and pigment use at Hohle Fels cave: Results of the first systematic review of ochre and ochre-related artefacts from the Upper Palaeolithic in Germany

**DOI:** 10.1371/journal.pone.0209874

**Published:** 2018-12-27

**Authors:** Elizabeth C. Velliky, Martin Porr, Nicholas J. Conard

**Affiliations:** 1 Institut für Naturwissenschaftliche Archäologie, Mathematisch-Naturwissenschaftlichen Fakultät, Tübingen, Germany; 2 Archaeology/Centre for Rock-Art Research and Management, M257, Faculty of Arts, Business, Law and Education, School of Social Sciences, The University of Western Australia, Crawley WA, Australia; 3 Institut für Ur- und Frühgeschichte und Archäologie des Mittelalters, ROCEEH—The Role of Culture in Early Expansions of Humans, Tübingen, Germany; 4 Department of Early Prehistory and Quaternary Ecology & Senckenberg Centre for Human Evolution and Quaternary Ecology, University of Tübingen, Schloss Hohentübingen, Tübingen, Germany; Institucio Catalana de Recerca i Estudis Avancats, SPAIN

## Abstract

Though many European Upper Palaeolithic sites document early examples of symbolic material expressions (e.g., cave art, personal ornaments, figurines), there exist few reports on the use of earth pigments outside of cave art–and occasionally Neanderthal–contexts. Here, we present the first in-depth study of the diachronic changes in ochre use throughout an entire Upper Palaeolithic sequence at Hohle Fels cave, Germany, spanning from ca. 44,000–14,500 cal. yr. BP. A reassessment of the assemblage has yielded 869 individual ochre artefacts, of which 27 show traces of anthropogenic modification. The ochre artefacts are from all Upper Palaeolithic layers, stemming from the earliest Aurignacian horizons to the Holocene. This wide temporal spread demonstrates the long-term presence and continuity of ochre use in a part of Europe where it has not been systematically reported before. The anthropogenic modifications present on the ochre artefacts from the Gravettian and Magdalenian are consistent with pigment powder production, whereas the only modified piece from the Aurignacian displays a possible engraved motif. The non-modified artefacts show that more hematite-rich specular ochres as well as fine-grained deep red iron oxide clays were preferred during the Gravettian and Magdalenian, while the Aurignacian layers contain a broader array of colours and textures. Furthermore, numerous other artefacts such as faunal elements, personal ornaments, shells, and an ochre grindstone further strengthen the conclusion that ochre behaviours were well established during the onset of the Aurignacian and subsequently flourished throughout the Upper Palaeolithic at Hohle Fels cave.

## Introduction

Hohle Fels (HF) cave has contributed significantly to our current understanding of the earliest culture associated with the first anatomically modern humans (AMHs) in Europe known as the Aurignacian (ca. 44,000–34,0000 cal. BP) [1]. The Aurignacian assemblage at HF includes the earliest known musical instruments in the form of flutes, personal ornaments, figurines, and the earliest female statuette dating to ca. 38,000 years BP [2–5] most of which are made from mammoth ivory. Less known in the HF assemblage is the presence of numerous ochre artefacts stemming from all Upper Palaeolithic (UP) periods. In this paper, we present the first systematic study of an ochre assemblage at HF cave and, more broadly, the first detailed analysis of an ochre assemblage from a Central European UP site through different time periods. HF in southwestern Germany presents a unique opportunity to observe diachronic change throughout the UP due to its well-established chronology and stratigraphy. Our recent reassessment of the HF excavated material yielded 869 individual ochre artefacts, 27 (3.1%) of which show traces of anthropogenic modification. The artefacts stem from all UP periods present at HF and were found over the course of excavations at the site from 1975–2018. Here, we report the qualitative characteristics of the HF ochre assemblage that hitherto have not been reported. These aspects include the variety of ochre types present, differences in visual characteristics such as colour and texture, and the types and range of modifications. We then discuss how ochre use changes throughout the UP and the behavioural implications of these use patterns on a local and regional scale.

## Overview of European and African research history

Ochre is a colloquial term frequently used by archaeologists in reference to any sediment, clay, or rock containing varying amounts of iron oxide or oxyhydroxide (generally, 2Fe_2_O_3_ and FeO) minerals [6]. It appears in sedimentary, metamorphic, and igneous contexts as most rock types contain varying amounts of Fe that are oxidised at variable rates in different geological settings [7]. Because of the different amounts of iron content in the material, the colours expressed vary from yellow and red to purple and brown. Various types of ochre can be heat-treated in order to alter their original colours directly, yet archaeological support of this behaviour is at this point limited (however, see [8, 9, 10]). Experimental studies show that the characteristics between heat treated and non-heat-treated ochre are subtle and can be difficult to differentiate [11].

The habitual exploitation of ochre in Pleistocene contexts is often cited to be related to cognitive complexity, syntactical language, and symbolically mediated behaviour [12–23]. Due to the antiquity of ochre in African sites, with the earliest examples stemming from contexts dating to ca. 300,000 years BP [24–27], a heavy emphasis is placed on researching ochre materials in these areas in relation to the emergence of behavioural modernity. The discussion of this topic is vast, with some authors supporting a primarily symbolic interpretation of ochre due to the sheer abundance at specific sites [25, 28], others cautioning against assuming such interpretations without proper investigation [29, 30], and others exploring the range of functional applications and geological varieties of ochre materials [31–37]. This latter functional perspective has shown ochre to be a useful material for tanning hides [38, 39], as an insect repellent and UVA/UVB shield [40, 41], and as an adhesive for weapon manufacture [36, 42–44]. Indeed, archaeological contexts from Sibudu Cave in South Africa provide support for ochre as a residue on lithics [43, 45] as well as in a mixture with a milk-based protein that could have been applied to skin or other surfaces [46]. In Europe, comprehensive reports of ochre assemblages have decreased within the last 20 years (however, see [47, 48, 49]). Though several reports from older excavations exist [50–53], their contextual and stratigraphic integrity is often not secured and becomes blurry over time and with multiple handlings of collections. Furthermore, often larger pieces or associated finds, such as ochre grindstones or artefacts “painted” with ochre [52, 54–57] are the primary focus while overlooking other artefacts and not providing comprehensive overviews. Comparatively, another earth pigment, manganese oxide, has been the subject of more intensive recent investigations due to its prevalence in Middle Palaeolithic contexts [58] and association with questions surrounding Neanderthal behavioural complexities [59–61]. Some ochre contexts are present in Middle Palaeolithic and Châtelperronian horizons [62–67]; however, modified manganese oxide nodules appear more frequently and were apparently the preferred pigment-producing material for our hominin cousins. Additionally, one may argue that compared to African ochre studies, relatively few European UP ochre assemblages are systematically studied to the same extent (see [68, 69]). There is also a comparative lack of experimental studies within the last 20 years of investigations of the range of ochre applications specific to European contexts (however, for example see [38, 70, 71]), though more recent work is published on the applications of manganese oxides [58, 59]. However, this focus on other pigments in the place of ochre is not due to a lack of ochre and other pigment producing artefacts in European UP archaeological sites [8, 48, 66, 69, 72, 73]. Here, we present a systematic overview of ochre and pigments from a Central European cave site, and discuss how this record fits into the network of cultural traits in the late Pleistocene.

### Hohle Fels cave and the Swabian Jura

#### Swabian Jura geological and archaeological context

The Swabian Jura (German: *Schwäbische Alb*) is a mountain range bordering the Danube River, located in the southern part of Baden-Württemberg (BW), Germany. This part of the BW region falls within the larger geological complex of the Jura Mountain range in Europe [74, 75]. The Swabian Jura consists of Jurassic limestones and is characterised by karstic landscape features such as caves, dry valleys, underground watercourses, and sinkholes [74, 76–78]. The landscape and geological formation of the Swabian Jura, and more specifically the region near HF is the result of extensive erosional processes and weathering since the Cretaceous period due to severe climatic fluctuations [79–81].

**Fig 1:**
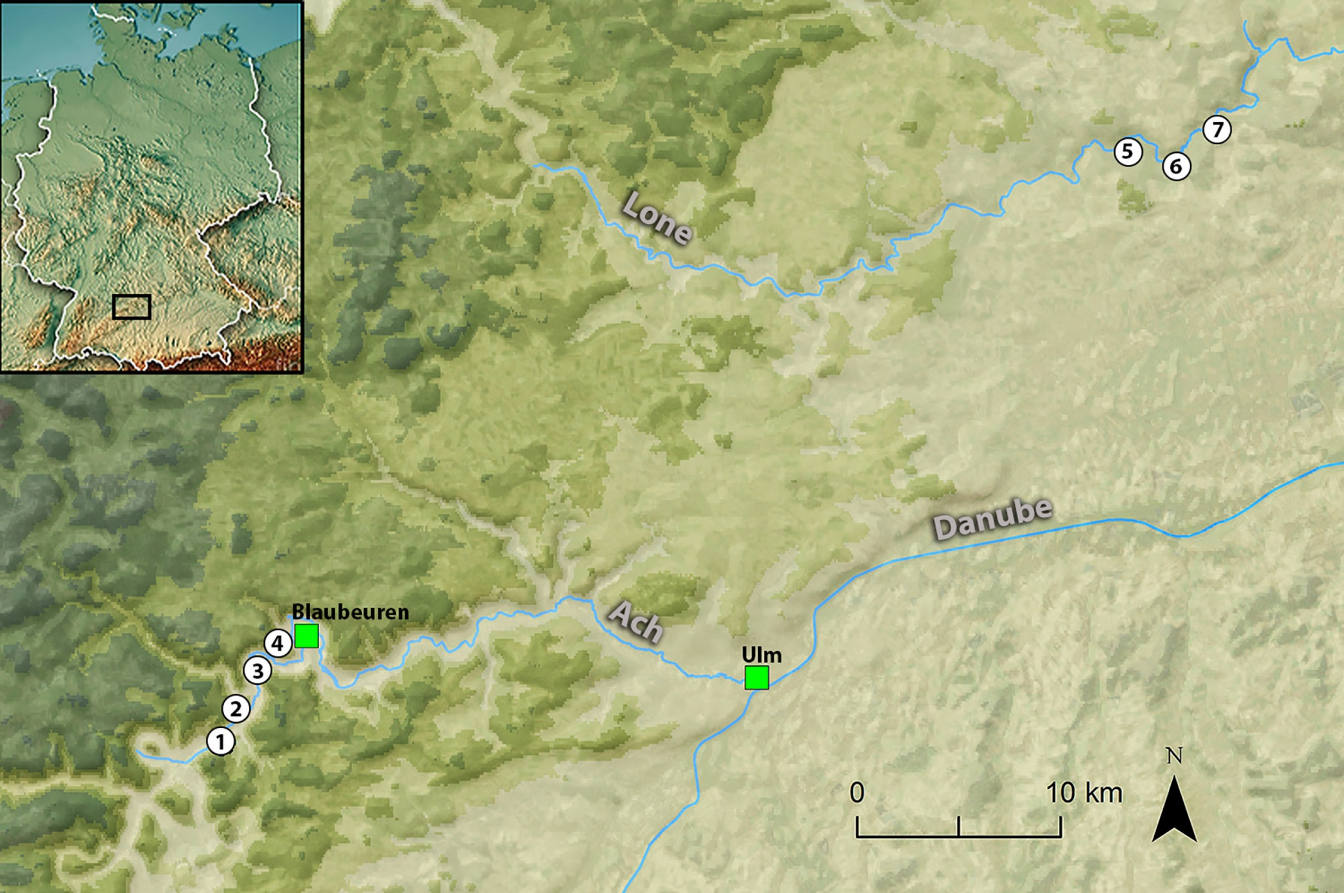
Map of southwestern Germany with the principal Upper Palaeolithic cave sites. Ach Valley: 1) Hohle Fels, 2) Sirgenstein, 3) Geißenklösterle, 4) Brillenhöhle; Lone Valley: 5) Bocksteinhöhle and Bockstein-Törle, 6) Hohlenstein-Stadel and Hohlenstein-Bärenhöhle, 7) Vogelherd.

The Swabian Jura is regarded as a critical area for understanding the early development of cultural and symbolic behaviours in human populations and contains numerous Middle Palaeolithic (MP) and UP sites [1, 82–84]. Most of the known MP and UP sites are located in two tributary valleys of the Danube (German: *Donau*), the Ach and Lone (German: *Achtal* and *Lonetal*) and include: Sirgenstein, Brillenhöhle, Hohle Fels and Geißenklösterle in the Ach Valley, and Vogelherd, Bockstein (Bockstein-höhle and Bockstein-Törle), and Hohlenstein (Stadel and Bärenhöhle) in the Lone Valley (Fig 1).

The assemblages from the Swabian Jura sites are among the earliest occurrences of the Aurignacian technocomplex in Europe, dated up to ca. 43,751 ± 654 cal. BP [3]. They are referred to by some as the *Swabian Aurignacian* due to their distinct artefact and tool composition [1, 85, 86]. Specifically, the *Swabian Aurignacian* is unique in Europe for its specimens of figurative art, musical instruments, blade and bladelet production as well as an organic tool industry [2, 3, 82, 87]. There is widespread agreement that the Swabian technocomplex is associated with AMHs who migrated into a favourable and apparently uninhabited region by way of the so-called Danube-Corridor around 45,000 years BP [82, 88].

In addition to the Aurignacian cultural sequence, the Gravettian (ca. 34,000–30,500 cal. yr. BP) and Magdalenian (ca. 16,500–14,500 cal. yr. BP) layers have also yielded a rich assortment of artefacts. The Gravettian boasts a large lithic assemblage including burins, Gravette points, and backed knives made from both local and non-local raw materials [89]. Numerous organic tools made from bone, antler, and ivory as well as personal ornaments made from various animal teeth and ivory characterise the assemblage [89, 90]. The Magdalenian lithic assemblage is similar to the Gravettian and also includes blades and bladelets, scrapers, and backed knives and points, to name a few [91] made from both local and non-local materials. Included in the assemblage are perforated animal teeth and freshwater snail shells, osseous tools of needles, harpoons, points, and notched rods, and jet “pendants” [91].

#### Hohle Fels cave

HF cave is 534 m above sea level, located between the modern-day towns of Blaubeuren and Schelklingen, some 17 km west of Ulm (Fig 1). HF is situated within a large, free-standing rock outcrop (i.e. tor, eng. or *Felsen*, ger.) of Jurassic limestone. The interior of HF is ca. 6000 m^3^ and is thus one of the largest caves in the Swabian Jura [77]. HF has an archaeological research history that extends back to the late 19^th^ century, beginning with Oscar Fraas’ excavations in 1870/71 with Theodor Hartmann [85, 92–94], though many of the finds went missing in World War II. Joachim Hahn and colleagues resumed research in 1973 with excavations at both Geißenklösterle and HF [93]. Here, Hahn implemented a systematic recording system and established the chrono-stratigraphy which is still the framework for the modern-day understanding of both sites. This work was continued in 1997 by Nicholas Conard in collaboration with Hans-Peter Uerpmann with the University of Tübingen.

**Fig 2:**
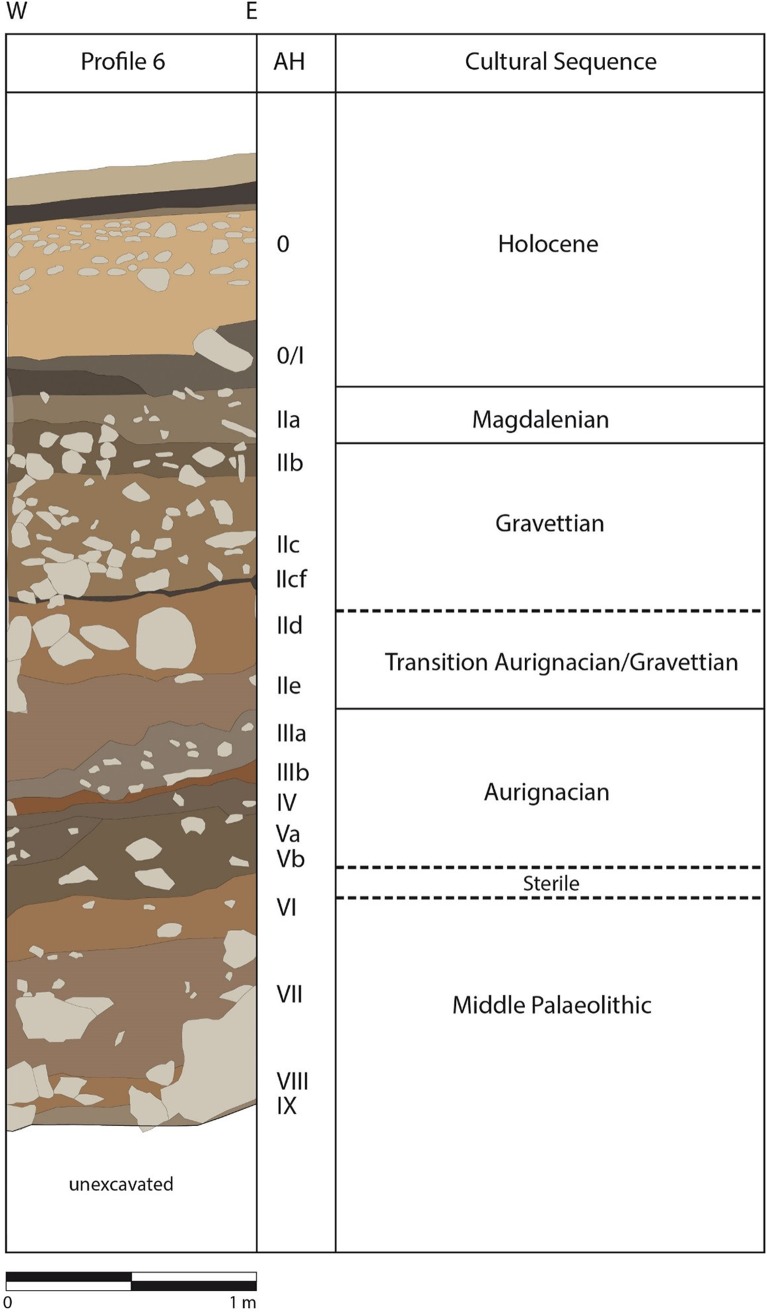
Cultural stratigraphy from Hohle Fels main profile. Schematic of main profile six at HF with archaeological horizons (AHs) in roman numerals and corresponding time period.

The current excavation, which is still ongoing, operates with 71 m^2^ spatial units in the northern apse of the cave close to the entrance, and bedrock has not yet been reached (Fig 2). The stratigraphy is organised based on litho- and archaeo-stratigraphic categories, resulting in the documentation of both geological horizons (GH) and archaeological horizons (AH). Although local variation occurs, the HF sediments are mainly composed of calcareous clay and locally phosphatic clay with less frequent inclusions of quartz, phosphatic grains, and organic material. The sediments throughout the sequence contain varying amounts of bone, lithic, and charcoal fragments intermixed throughout [76–78].

The three-metre deep HF stratigraphy is divided into six major archaeological horizons assigned to the Magdalenian (ca. 16,500–14,500 cal. yr. BP), Gravettian (ca. 34,000–30,500 cal. yr. BP), Aurignacian (44,000–34,000 cal. yr. BP), and MP (>44ka BP) layers (see Table 1 for uncalibrated radiocarbon dates). These are further sub-divided into finer excavation units (Fig 2), starting with the uppermost Magdalenian layers AH I and AH IIa, both dated to around 16 ka cal. BP [91]. The Magdalenian layers show some internal mixing (I and IIa) as well as with the overlying Holocene layers [91]. The most recent Gravettian layer AH IIb also contains some Magdalenian artefacts, especially in the northwestern units, and may represent a period of post-depositional mixing [95]. The layers IId and IIe are brief and document a short, if at all existing, occupational hiatus between the Gravettian and Aurignacian (ca. 34,500–32,500 cal. yr. BP) [96, 97]. The Aurignacian complex contains sequences AH IIIa-b, IV, Va, Vaa-ab, and Vb, all of which have yielded many figurative art and symbolic artefacts as well as a rich lithic industry [91, 95, 98].

**Table 1:** Dates for the Hohle Fels Upper Palaeolithic. Uncalibrated radiocarbon dates for corresponding archaeological horizons at HF. Period abbreviations are: M = Magdalenian, G = Gravettian, A/G = Aurignacian/Gravettian transition, A = Aurignacian.

AH	Period	Uncal. date	References
I	M	13,240 ± 110–12,506 ± 32	Hahn, 1995; Taller, 2014
IIa	M	13,350 ± 140–12,520 ± 130	Housley et al., 1997
IIb	G	28,350 ± 220–28,170 ± 180	Hofreiter et al., 2007
IIc	G	29,500 ± 650–26,000 ± 360	Hahn, 1995; Housley et al., 1997
IIcf	G	27,970 ± 140–27,030 ± 240	Conard, 2003
IId/IIe	A/G	30,640 ± 190–28,060 ± 170	Conard and Bolus, 2003; Conard and Moreau, 2004
IIIa/b	A	29,990 ± 330–29,710 ± 210	Conard and Bolus, 2003; 2008
IV	A	33,090 ± 250–30,110 ± 210	Conard and Bolus, 2003; 2008
Va	A	35,710 ± 340–31,750 ± 260	Conard and Bolus, 2003; 2008
Vb	A	40,000 ± 500–31,290 ± 180	Conard, 2009

#### Previously recorded ochre and ochre related artefacts

In the years 2009 and 2010, five different ochre artefacts were recovered from Magdalenian contexts at the site, all bearing different forms of anthropogenic modification [99, 100]. Four of the five pieces are hematite, a purple to silver iron oxide (Fe_2_O_3_) that produces deep red streaks and is often found as a red pigment in archaeological contexts [6, 8, 33, 67, 72, 101, 102], the other piece is classified as a “red chalk” (Find # 102.555.1). This find contains scoring incisions on all four of its worked surfaces, forming a “pencil” or “crayon” shape (S1A Fig). A specular hematite artefact (Find # 102.630.1) was ground on two sides forming two faceted surfaces which converge to a point, though the tip is broken off (S1A Fig). The remaining three pieces, referred to as the *Rondelle* artefacts [100], appear to be of the same specular hematite material and were rounded into a circular shape with a perforation in the centre (S1A Fig). Two of these pieces refit together (Find # 110.1104.1 & 110.992), and it is possible that the third piece (Find # 110.434.1) is part of the same or an entirely different artefact. The exact purpose of this worked piece is unknown, though similar forms of disc-shaped artefacts made from shale and jet were found in Switzerland and the Czech Republic as well as HF [91], though they are thought to have had a functional purpose in the construction of habitation structures [103, 104]. Other *Rondelle* made from bone have been found in Magdalenian contexts in Southwest Europe and later on in Central Europe, indicating that this style may have been trans-regional [103, 105].

Excavations in 1998 uncovered a painted fragment of the same Jurassic limestone of the interior of the cave. This piece might have originally been attached to the cave wall [106]. In total, seven painted rocks, including limestone and dolomite materials, have been found in the Magdalenian layers [99, 100, 106–110]. All of these pieces contain traces of red ochre arranged in a series of painted rows of dots. The rows occur in pairs and range from one to three pairs on each stone (S1B Fig). Two of the limestone fragments are rounded and do not refit with any of the other pieces or the cave wall. Their material and lack of refit suggest that instead of sourcing from the interior of the cave, it is possible that they are river cobbles from the Ach (located directly outside of the cave) or the Danube rivers. The stones come from layers AH I and IIad and contain some of the better-preserved series of rows of dots on the painted stone assemblage. It is likely that the original paintings were more substantial and may have contained numerous designs, whether they were stylistically similar or not [69]. The lack of preservation of painted artefacts, whether it be parietal or portable art, may be due to the deterioration of the cave walls in this Karstic region which are subject to expansion and retraction during climatic warming and cooling phases, as well as erosional processes [111].

In addition to the painted stones, faunal elements with traces of ochre from the Magdalenian and Aurignacian levels of HF also constitute part of the assemblage (S1C Fig) [107]. One long bone (Find # 14.69) displays two large oblong smudges of red ochre. A fragment of a reindeer cranium (Find # 89.48) contains traces of red ochre powder on the interior. One temporal fragment of a cave bear (Find # 29.1484.13) with traces of red ochre residue on the interior comes from the Gravettian layers [69]. In summation, the previously recognized modified ochre artefacts, as well as the artefacts exhibiting visible traces of anthropogenically applied red residues, suggest that ochre behaviours were well in place at the site by the late Pleistocene.

## Materials and methods

The term *ochre* is used quite liberally in the archaeological community in reference to any earth-derived colouring material, often showing red, orange, yellow, purple, brown, or black hues, which can be manipulated into a pigment [23, 28, 35, 112–116]. The designation of ochre also implies the intentional recognition, acquisition, and transportation of the material [117]. Thus, in the archaeological sense, the term *ochre* does not only refer to minerals in the landscape that are red but also minerals that were interacted with and collected by hominin species. We use the term *ochre artefact* or *ochre piece* in order to specify that these items are archaeological materials recorded and collected from excavations at HF, whether they be unmodified iron oxide nodules or patches of red iron-rich sediment. Other terms used throughout this paper include *anthropogenically modified* or simply *modified ochre* which refers to ochre artefacts that were further altered by hominins and contain visible traces from these physical interactions. We emphasise the ochre data and results from the Aurignacian, Aurignacian/Gravettian transition (A/G Trans), Gravettian, Magdalenian, and, to a lesser extent, Holocene ochre artefacts. All artefacts catalogued as RO (*Rötel* or red ochre) or HA (*Hämatit* or hematite) recorded as single plotted finds found *in-situ* during excavation, bucket sediment finds, and coarse fraction finds were included in this in-depth analysis. The entire HF assemblage, including the ochre materials, is housed and curated at the University of Tübingen in the *Institut für Naturwissenschaftliche Archäologie* (INA) and the Department of Early Prehistory and Quaternary Ecology in the *Schloss Hohentübingen*, and therefore no special permissions were required for analysing the artefacts. The archaeological assemblage was reinvestigated by the primary author (EV) between the years 2015–2018, and this revealed hundreds of previously unidentified and non-categorized ochre pieces. The ochre artefacts were catalogued under a unique system (specimen numbers HF Ochre 1–955) in order to avoid duplicates for rediscovered ochre artefacts and are correlated to the general HF archaeological database. No permits were required for the described study, which complied with all relevant regulations.

One category frequently collected at HF is OK (*Ocker* or yellow ochre). Even though yellow ochre artefacts outnumber red ochre artefacts (n = 1,007 for plotted single finds alone), the vast majority of these yellow ochre pieces are likely weathered limestone naturally occurring in the cave. Therefore, we did not include the yellow ochre artefacts as there was no way to distinguish between naturally occurring and anthropogenically transported yellow ochre at the site without using chemical or mineralogical techniques. The exclusion is not meant to suggest that yellow ochre was not collected and brought to the site or perhaps even heat treated to alter the colour as seen in other archaeological sites [9–11, 72]. This extensive work of chemically and mineralogically comparing the yellow ochre artefacts to the naturally occurring weathered limestone fragments is therefore left for future analysis but does not form part of the current research project.

In our revised ochre artefact database from HF, each piece was given a unique identifying code and was classified by a series of qualitative characteristics (Table 2). Each of the artefacts in this database was macro- and microscopically examined for traces of modification using a Euromex binocular microscope with 10–30 x magnification. Macroscopic variables were determined using a combination of natural and artificial light. If pieces were determined to bear traces similar to anthropogenic modifications, they were further examined in more detail using a Zeiss Discovery V8 Stereomicroscope. Photographs were taken with either the Zeiss or a Keyence VHX500 digital microscope. CIELAB colour measurements were conducted on digital images taken with the Keyence VHX500 using the same fixed light setting and then measured in Adobe Photoshop. Similar protocols for measuring colour using CIELAB values have also been incorporated into previous qualitative studies of ochre and pigments [31, 32, 118]. While the resulting colour values refer to the true calibrated surface colour of the ochre piece, it is our opinion that the internally consistent CIELAB values we produced are indeed representative of the original surface colour.

**Table 2:** Ochre characteristics for Hohle Fels artefacts. Descriptive variables for ochre and ochre-related artefacts.

**Category—Ochres**	**Variables**
Size	L x W x D (mm), weight (g)
Rock types	Hematite, sandstone, iron oxide, clay, red sediment, siltstone, specularite
Characteristics	Micaceous, oolitic, porosity
Texture	Clay, silt, sand (mixtures)
Colour, second colour	Purple, red, orange, yellow, brown (dark/light)
Modified	Striations, micro-striations, score marks
**Category—Residues**	**Variables**
Size	L x W x D (mm), weight (g)
Material	Stone, mammoth ivory, limestone, bone, tooth, shell, fossil
Colours	Artefact colour, residue colour, # of colours
Residue	Applicator, grinding stone, personal ornament, painting, mixture, container
Residue origin	Direct application, indirect staining, natural

Our classification of ochre modifications builds on protocols established in previous ochre studies in order to maintain consistency within the research field and to allow for future cross-comparisons between sites and ochre assemblages [23, 31, 119–121]. Qualitative characteristics such as texture, colour, rock type, and size of the ochre artefacts are described based on a system created by Hodgskiss (119); however, these traits are common descriptive variables used for more qualitative ochre analyses elsewhere [27, 31–33, 120, 122–124]. Our HF protocol (for details see Table 2) allows us to characterise grinding, rubbing, scoring or more specifically incisions or engraving striations, micro-striations, rounding and faceting.

### Physical characteristics

Our examination and description of the HF ochre assemblage are inspired by and adapted from methodologies employed by researchers investigating Middle Stone Age (MSA) ochre from South Africa [31, 113, 120, 123], with some alterations made by the primary author. Since the HF ochre artefacts vary considerably from MSA assemblages, several categorical descriptions were altered to accommodate local characteristics of the HF assemblage. These descriptions are not meant to apply to all European assemblages. They do, however, provide a starting point for further discussion on how to apply commonly used terms and techniques in ochre research to a geographical region where research on pigments in the UP is comparatively less emphasised. The characteristics, based on Hodgskiss and Wadley [123], are as follows:

1. *Rock type*. Each artefact classifies to one of seven rock types (hematite, sandstone, specularite, clay, red sediment, siltstone, iron oxide) based on visual examination. These rock type categories are purposefully broad, as the artefacts show a high degree of heterogeneity, preventing a precise mineralogical classification. *Hematite* is classified by a visually purple colour with a fine-grained texture, a red to dark red streak and often containing mica inclusions. *Specularite* is generally extremely dark purple to black and contains a high amount of mica inclusions. *Sandstone* is characterised by a redder colour and sand-sized granular texture, *siltstone* and *clay* are differentiated based on grain size. *Red sediment* was any ochre material recovered not in a solid form. *Iron oxide* was treated as an “other” category when could not be classified into one of the aforementioned categories. Quartz and mica inclusions are noted, as well as the presence of porous and oolitic textures.
2. *Grain Size*. Individual pieces were given approximate texture classifications: clayey (diameter <2 μm), silty (diameter <50–2 μm) and sandy (diameter <2000–50 μm) [125]. In most cases pieces contained a combination of grain sizes, so a descriptive combination of different textures was employed based on the primary and secondary textures (e.g. clayey/silty, silty/sandy).
3. *Colour*. Artefacts were assigned a subjective category based on their external colour including purple, red, pink, orange, yellow, and variations of these including dark or light. We also recorded streak colour as it is likely that pigment colour played a more significant role than the exterior surface colour of the physical piece. Streak tests were conducted on an unpolished white ceramic plate and were subsequently photographed; Adobe Photoshop CS6 was used to measure the average CIELAB colour values in an 11 x 11-pixel area.

In general, ochre found at archaeological sites tends to exhibit hardness levels between three and five [28, 35, 113, 119, 120, 123]. Given the small size (<10 mm) of many of the HF ochre artefacts (n = 512), it was not possible to systematically measure hardness. Colour streak tests were conducted on the ochre artefacts in order to identify the colour of the produced powder. The most obvious example is pure hematite pieces, which commonly exhibit a silvery or greyish shimmer on their exterior, and produce a dark red streak or powder. This form of analysis is semi-destructive but provides essential information on the pigment colour of the artefact, which likely played a larger role in ochre selection than exterior colour. Each of the ochre pieces large enough to hold (≥ 5 mm) underwent a streak test on an unpolished ceramic plate. The streaks were grouped based on their stratigraphic temporal assignment: Aurignacian, A/G transition, Gravettian, and Magdalenian. All of the ceramic plates were photographed under the same magnification and light conditions using a Keyence VHX500 digital microscope. The images were taken in TIF format and exported to Adobe Photoshop CC 2016. The colour of each streak was measured within a 3-dimensional colour space, CIELAB, or International Commission on Illumination (CIE) L*a*b* colour space using an 11x11 pixel average method. This method for measuring colour has been applied to other archaeological ochres with the intention of studying behavioural patterns relating to colour selection and preference [31, 32], and allows for a more quantitative assessment of the range and distribution of colours that ochre artefacts can produce.

### Anthropogenic modifications

According to previously conducted ochre studies [47, 113, 120, 124], an evaluation of the frequency and nature of surface modification can allow inference on the nature of anthropogenic modifications. After visual examination, the HF ochre artefacts were classified into three categories of modification: *modified*, *possibly modified*, or *non-modified*. The presence of modifications was established based on the occurrence of certain traits including striations, micro-striations, faceting, polish, and scoring. These definitions are based on Hodgskiss’ [112] initial experimental work on ochre use-wear patterns and are as follows:

1. *Grinding*. Defined by the presence of multiple groups of parallel striations or grooves. Micro-striations may also be present, and extensively ground pieces may become faceted. This type of modification is caused by rubbing an ochre piece on a hard surface and was likely done to create a concentrated patch of pigment powder. Profile shapes of grooves can vary based on the surface morphology of both the ochre piece and the grindstone.
2. *Rubbing*. Defined by the presence of micro-striations, polish, and the removal or smoothing of surface morphological features. Rubbing is also defined as soft-surface grinding and indicates a direct application of an ochre piece to a soft surface such as human or animal skin. Rubbing can also coincide with grinding ochre pieces, as post-ground surfaces can contain much powder and could have been directly applied to soft surfaces to create a streak of colour. Traces of rubbing can also be created post-depositionally and are differentiated in this research based on other post-depositional characteristics (e.g., scuff or scratch marks), or with the presence of other forms of anthropogenic modification. If no definitive assertion could be reached, then the piece was categorised as *possibly modified*.
3. *Scoring*. Refers to a deep incision of cut as applied by a tool or device, likely a lithic or bone fragment. Profile shapes of score marks vary greatly depending on depth and precision of the incision, often contain micro-striations within the incision, can show varying depths in the incisions, and can contain frayed ends due to multiple-stokes in an incision. Several score marks can form an *engraving* which shows an intentional shape or form designation.
4. *Faceting*. A result of intensive grinding which changes the shape of an artefact so substantially that the ground surface is entirely flat. The surface often contains striations or micro-striations, however rubbing or post-depositional processes can erase these.
5. *Rounding*. A general shaping of an artefact so that the outer edge shows a circular profile. Striations or micro-striations are often present on the rounded surface. Rounding can refer to both convex and concave edges.

Ochre artefacts can also exhibit evidence for more than one form of anthropogenic modification. Rounding is a form of grinding, faceted surfaces are often the result of grinding, and scoring can occur on faceted surfaces. Rubbing or smoothing can decrease the severity of profile shapes or striations, and also alter edge shapes on the artefacts. For each category, profile shapes of striations, orientations, surface morphology, the presence of polish, and presence of modern modifications (scuff marks, scratches) were noted.

### Ochre-related artefacts (ochre residues)

In addition to the identification and cataloguing of individual ochre pieces, artefacts with traces of what appeared to be red ochre residues were collected and categorised in order to investigate broader patterns of ochre use. Many of these artefacts, such as faunal elements, perforated ivory beads, and freshwater snail shells, were visually identified and separated during the reassessment of the HF ochre assemblage. Dr. Susanne Münzel of the University of Tübingen previously identified other artefacts during various analyses of the faunal assemblage. We categorised these by similar variables used for the ochre artefacts such as artefact type, size, period, and type of material, the colour of residue, the presence of residue, possible use, and final decision or designation of the cause of the residues. These are defined below. However, a breakdown of the other qualitative variables is described in Table 2.

1. *Direct application*. This category describes artefacts that based on all visual evidence were likely intentionally and directly coloured with red ochre. Examples include the previously discovered painted limestone pieces [69, 99, 100, 110].
2. *Indirect colouring*. This category contains artefacts that are covered with ochre, potentially during ochre powder processing, or by indirectly colouring as a result of other anthropogenic processes like rubbing against hides or other surfaces. It is also possible that the colouring of artefacts in this category is a result of sediments rich in anthropogenically derived ochre powder. Examples include freshwater snail shells or faunal elements with a visible layer of ochre powder.
3. *Natural*. The colouring and staining of the object is likely the result of natural cave processes, staining from geogenic cave sediments, non-anthropogenically derived iron oxide or other post-depositional processes. *Natural* colouring differs from *indirect colouring* by considering the sedimentary micro-context of the artefact, i.e. staining was considered natural when the colouring agent was part of the sedimentary matrix. Examples include limestone pieces with red surfaces that likely result from oxidisation, and faunal elements where the colouration is part of the material matrix and not an external application of ochre powder.

## Results

**Fig 3:**
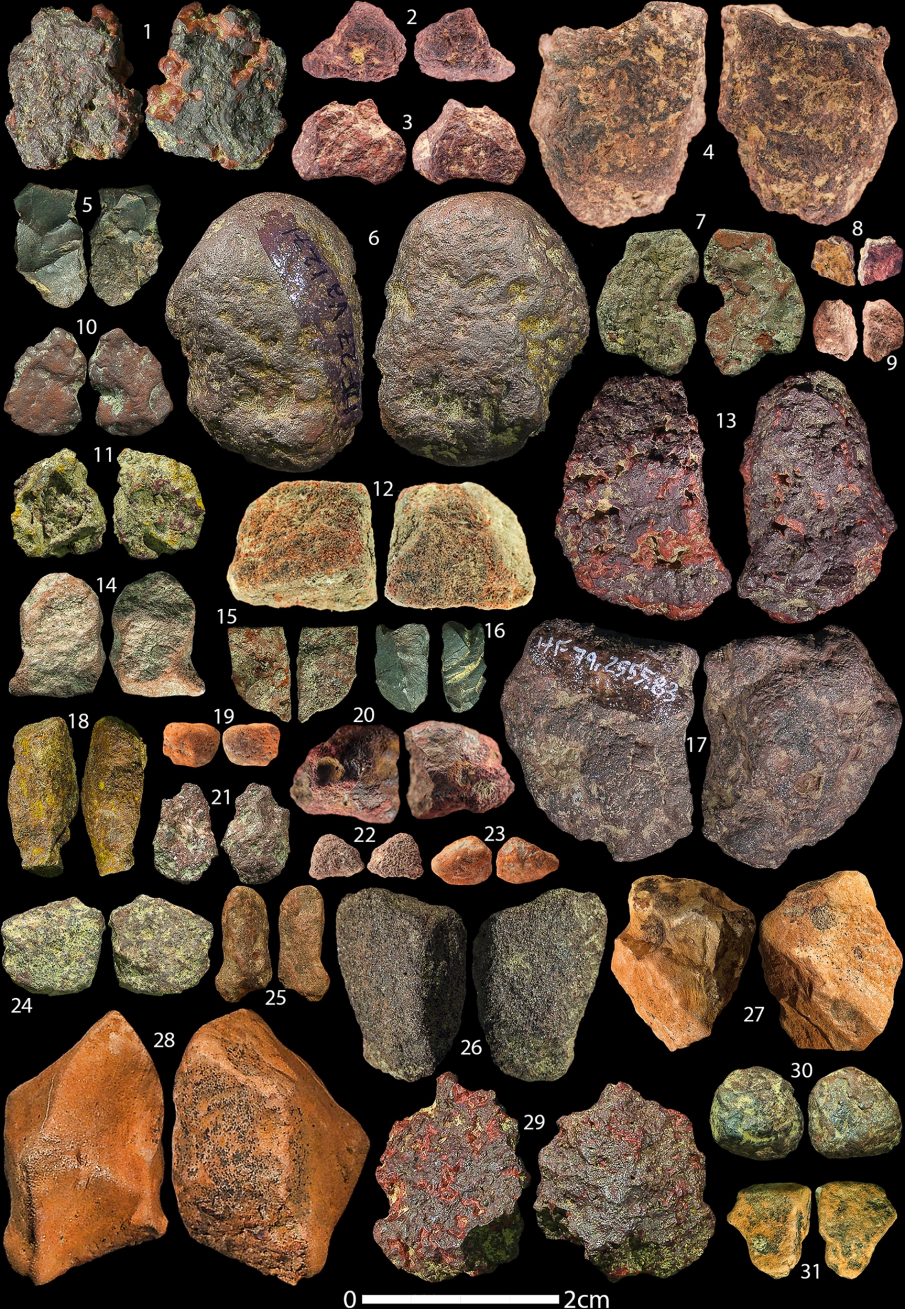
Hohle Fels Aurignacian and A/G transition ochres. Selection of unmodified ochre artefacts from the Aurignacian and A/G transition at HF. Numbers correspond to Table A in S1 Table.

**Fig 4:**
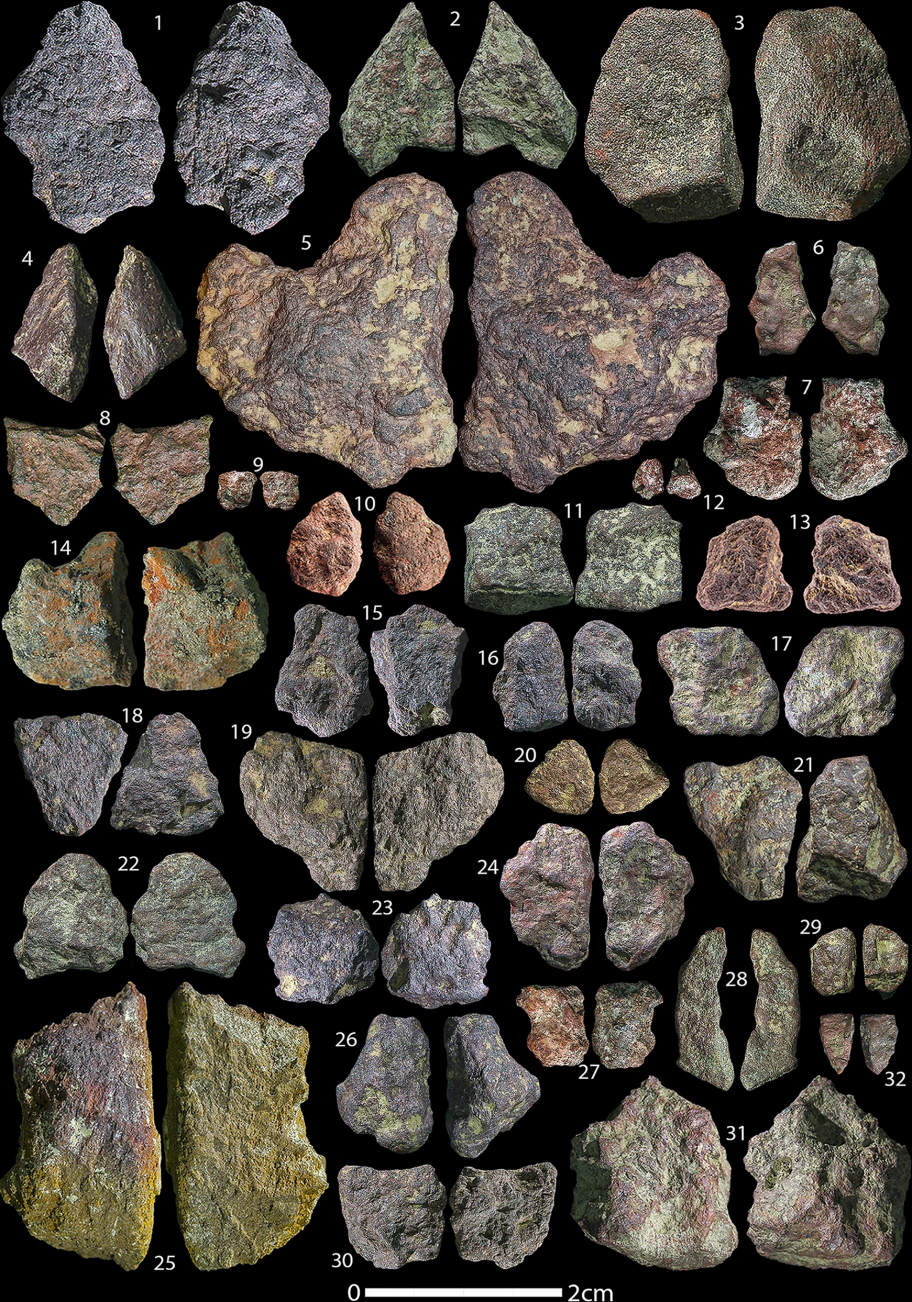
Hohle Fels Gravettian ochres. Selection of unmodified ochre artefacts from the Gravettian layers at HF. Numbers correspond to Table B in S1 Table.

**Fig 5:**
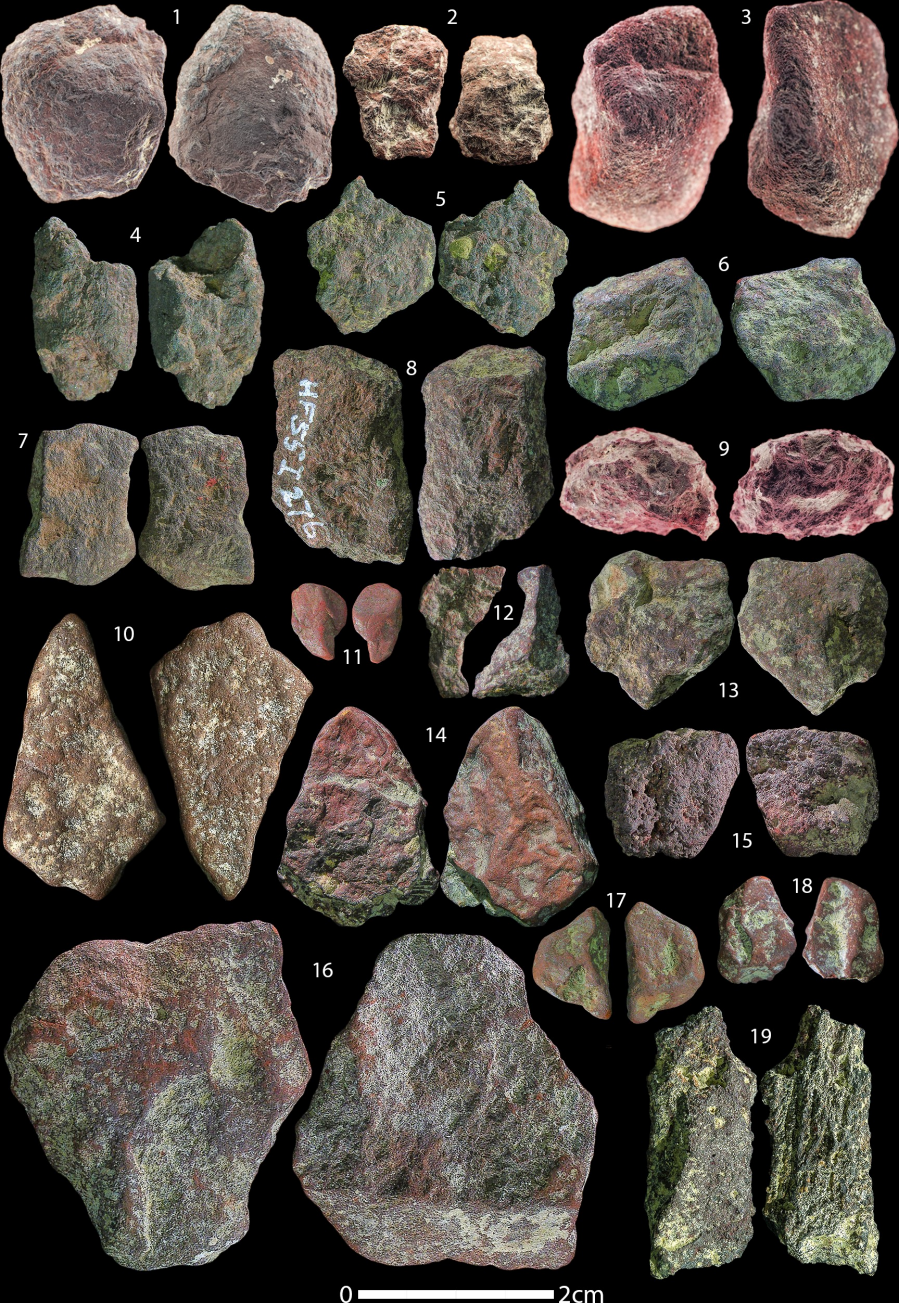
Hohle Fels Magdalenian ochres. Selection of unmodified ochre artefacts from the Magdalenian layers at HF. Numbers correspond to Table C in S1 Table.

Our reassessment of the HF ochre assemblage yielded a total of 869 individual ochre pieces, including artefacts from the Holocene, with a total weight of 925.768 g (average 1.07 g). A breakdown of the total number of ochre artefacts and modified pieces in all time periods from HF is summarised in Table 3. 27 artefacts show definite signs of anthropogenic modification (3.1% of the assemblage) and another 21 show possible traces of anthropogenic modification (2.4% of the assemblage). The Aurignacian layers yielded the most ochre artefacts with 371 (Fig 3), yet it contains only one anthropogenically modified artefact and two possibly modified pieces. The Gravettian layers contain 278 individually recorded ochre artefacts (Fig 4), of which seven show traces of anthropogenic modification and two of which are possibly modified. The Magdalenian layers yielded fewer ochre artefacts (n = 164) (Fig 5), yet this period has the most modified (n = 17) and possibly modified (n = 14) artefacts.

**Table 3:** Hohle Fels ochre artefact totals. Final number of ochre pieces, modified ochre artefacts, and possibly modified ochre artefacts from HF.

	Holocene	Magdalenian	Gravettian	A/G Trans	Aurignacian	Total
**Total**	**21**	**164**	**278**	**35**	**371**	**869**
Unmodified	17	133	269	34	368	821
Modified	1	17	7	1	1	27
Possibly Modified	3	14	2	0	2	21

Below, we will focus further on the in-depth analyses of UP cultural periods: the Aurignacian, the A/G transition, the Gravettian, and the Magdalenian. Though the Holocene layers are likely mixed with Magdalenian layers and perhaps contain numerous Magdalenian ochre artefacts, their stratigraphic integrity cannot be assured.

### Rock types

Table 4 displays a detailed breakdown of the qualitative ochre characteristics per cultural period in the UP at HF. In the entire HF assemblage (modified, possibly and non-modified) from all periods, hematite is the dominant rock type (48.4%), followed by sandstone (27.8%) and iron oxide (17.8%). A period comparison shows that this pattern is mainly present in the Magdalenian and the Gravettian non-modified ochre assemblages, however, in the Gravettian iron oxide is the second most abundant (16.8%) followed by sandstone (13.9%). This pattern changes slightly for the Aurignacian non-modified ochres, where the dominant rock type is sandstone at 45.3%, followed by hematite (25.7%) and iron oxide (18.2%). When looking at only the modified ochre, hematite is also the favoured rock type (74%) followed by iron oxide (11%).

**Table 4:** Numbers and percentages of measured qualitative characteristics on the Hohle Fels ochre assemblage. Highest values are indicated by green and lowest values indicated by red.

Ochre qualitative characteristics by period		Aurignacian		A/G transition		Gravettian		Magdalenian		Total	
		n = 371	%n	n = 35	%n	n = 278	%n	n = 164	%n	n = 848	%n
**Rock type**	Hematite	93	25.7%	9	31.0%	185	67.5%	112	70.0%	399	48.4%
	Iron oxide	66	18.2%	16	55.2%	46	16.8%	19	11.9%	147	17.8%
	Sandstone	164	45.3%	7	24.1%	38	13.9%	20	12.5%	229	27.8%
	Clay	5	1.4%	1	3.4%	3	1.1%	7	4.4%	16	1.9%
	Siltstone	4	1.1%			1	0.4%	4	2.5%	9	1.1%
	Deg. Limestone	2	0.6%	1	3.4%					3	0.4%
	Specularite	3	0.8%							3	0.4%
	Red sediment	34	9.4%	1	3.4%	5	1.8%	2	1.3%	42	5.1%
**Grain Size**	Clayey	37	10.2%	3	10.3%	31	11.3%	23	14.4%	94	11.4%
	Clayey/Silty	19	5.2%	1	3.4%	75	27.4%	25	15.6%	120	14.5%
	Silty	45	12.4%	10	34.5%	23	8.4%	25	15.6%	103	12.5%
	Silty/Sandy	31	8.6%	8	27.6%	75	27.4%	50	31.3%	164	19.9%
	Very fine sand	34	9.4%	4	13.8%	24	8.8%	17	10.6%	79	9.6%
	Fine sand	183	50.6%	8	27.6%	45	16.4%	21	13.1%	257	31.2%
	Medium sand	22	6.1%	1	3.4%	5	1.8%	3	1.9%	31	3.8%
**Colour**	Pink	5	1.4%			7	2.6%	3	1.9%	15	1.8%
	Light red	61	16.9%	3	10.3%	28	10.2%	11	6.9%	103	12.5%
	Red	91	25.1%	4	13.8%	26	9.5%	22	13.8%	143	17.3%
	Dark red	48	13.3%	5	17.2%	9	3.3%	14	8.8%	76	9.2%
	Brick red	40	11.0%	1	3.4%	7	2.6%			48	5.8%
	Rust	6	1.7%	5	17.2%	6	2.2%	1	0.6%	18	2.2%
	Light purple	1	0.3%	1	3.4%	6	2.2%	5	3.1%	13	1.6%
	Purple	18	5.0%	5	17.2%	124	45.3%	48	30.0%	195	23.6%
	Dark purple	86	23.8%	7	24.1%	61	22.3%	58	36.3%	212	25.7%
	Yellow	2	0.6%	1	3.4%					3	0.4%
	Orange	1	0.3%	3	10.3%	2	0.7%	1	0.6%	7	0.8%
	Brown	8	2.2%			1	0.4%	1	0.6%	10	1.2%
	Black	4	1.1%			1	0.4%			5	0.6%
**Mica**	Present	41	11%	5	14.2%	128	46%	47	28.6%	221	26%
**Oolitic**	Present	44	12.2%			7	2.6%	2	1.3%	53	6.4%

### Textures

The textures of the ochre artefacts from HF correlate well with their rock types. For example, hematite pieces are often of a silty texture with varying sandy or clayey mixtures. This textural affiliation is present in the A/G transition, Gravettian and Magdalenian, all of which contain about 30% silty-textured ochres for modified, possibly, and non-modified ochres (see Table 4). The texture preference changes for the Aurignacian, as the majority of the ochre artefacts are sandstones with a fine-grained sand texture constituting 50.6% of the assemblage. All of the time periods contained varying amounts of sandstone and iron oxides, which contributed to the most frequent texture type of all time periods and modification types combined being fine-grained sand (31.2%).

### Colours

The surface colour of the ochre artefacts also corresponds to the frequency of certain rock types. Dark purple is the most common colour designation for the total assemblage at 25.7%, followed by purple at 23.6%, which both correspond to the most frequent rock type for the total assemblage, hematite. The most common colour designation for the Aurignacian is red (25.1%), with dark purple as the second most frequent colour in the Aurignacian at 23.8%. The colour preferences shift to dark purple beginning with the A/G transition (24.1%) and continuing throughout the Magdalenian (36.3%), with the Gravettian containing a majority of purple ochre artefacts at 45.3%.

**Fig 6:**
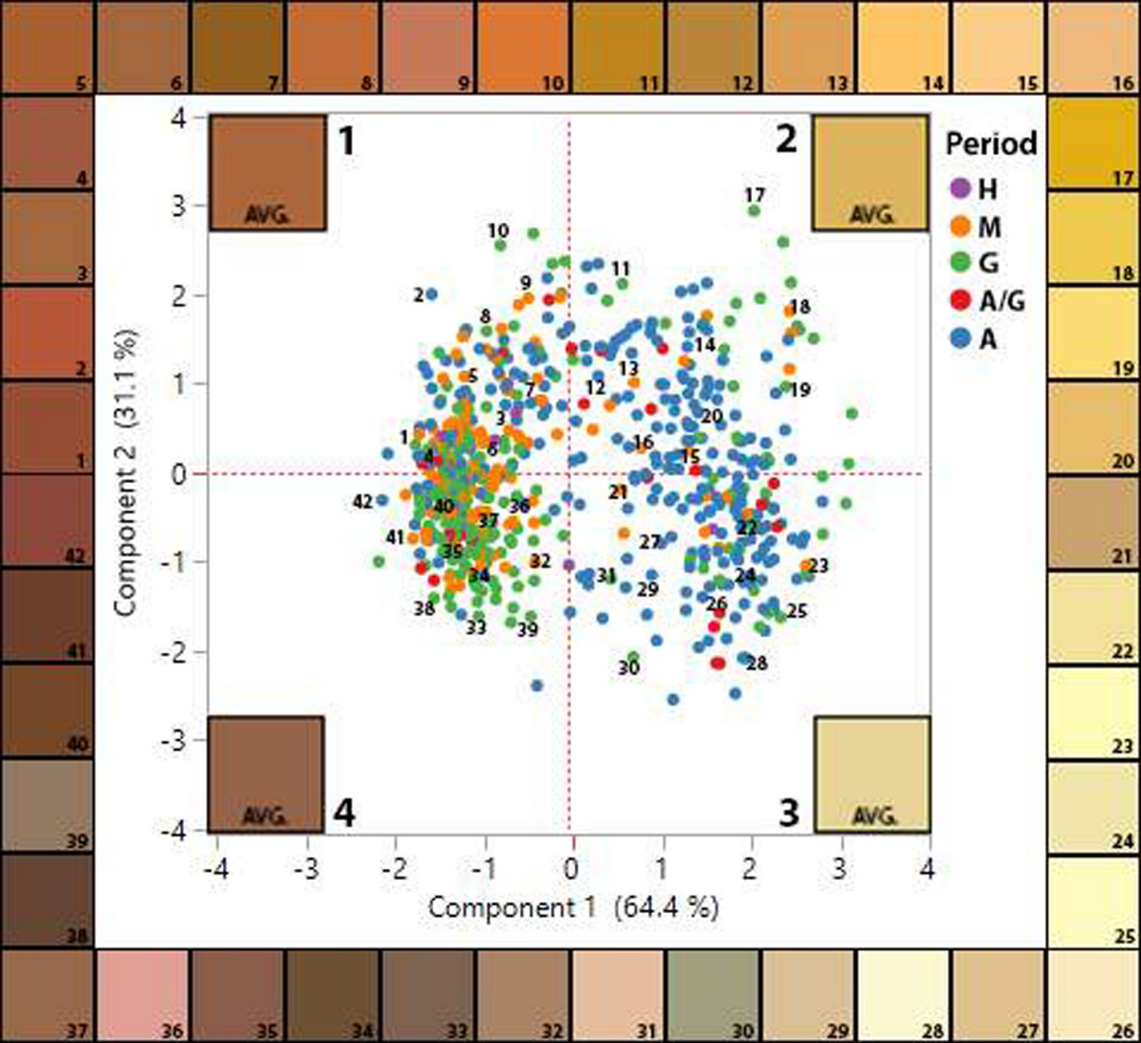
PCA plot showing first two components of CIELAB colour values on ochre streak tests from Hohle Fels ochres. Average CIELAB colour of the quadrant shown in corner of the quadrant. Specific point colours shown around the border of the plot with numbers in the PCA chart indicate corresponding data points. Data points are organized by period as shown on legend: H = Holocene, M = Magdalenian, G = Gravettian, A/G = Aurignacian/Gravettian transition, A = Aurignacian.

**Fig 7:**
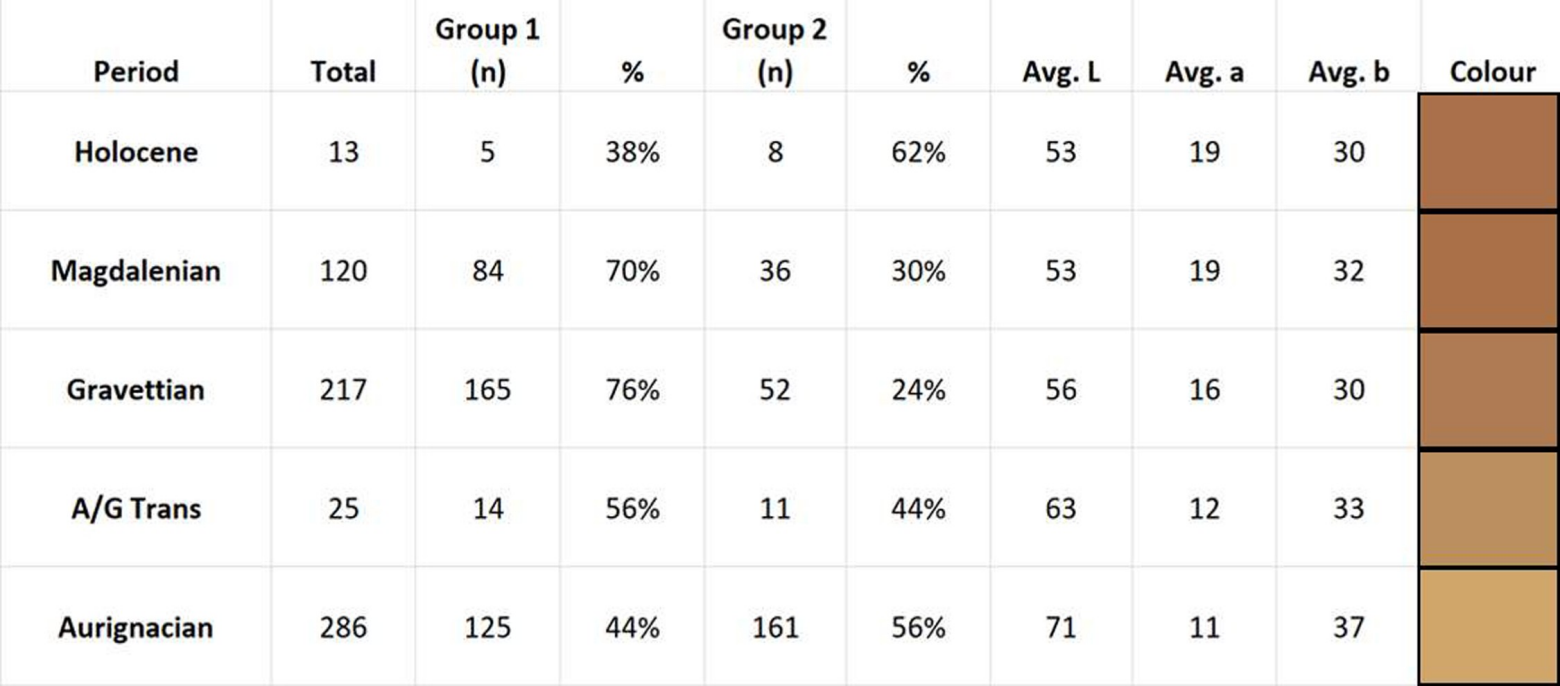
Hohle Fels ochre streak data per time period. Total number of streaks analysed as well as totals for corresponding cultural periods. The average values for L*a*b* per period are also displayed, as well as the corresponding colour for the averaged values.

For the streak colour tests, we tested 694 individual ochre artefacts. Each of the corresponding CIELAB values was statistically analysed using principal components analysis on correlations (PCA) in order to observe the total amount of variance within the assemblage (Fig 6). The first two principal components account for 95.5% of the total variation in the dataset (eigenvalue: 1.9328). Several notable observations can be made. The data generate two clusters separated along the *y-*axis, which account for differences in lightness (L*) and green-red components (a*) of the ochre pieces. The blue-yellow component (b*) also accounted for some variation, especially in quadrant two. The separation of groups along the *y*-axis also show some distinction in the temporal spread of the ochre streaks. Group 1 (quadrants one and four) contains 70% of the Magdalenian ochres tested and 76% of the Gravettian ochres tested, while Group 2 (quadrants two and three) contains 56% of the Aurignacian ochres, indicating visible differences in the lightness and green-red values of the colour streaks between the earlier and later time periods. This is further indicated by comparing the average LAB colour for each period (Fig 7), showing a darker colour and lower L* (lightness) value for the later time periods and high L* values displaying lighter colours in the earlier time periods.

The measurement of colour using CIELAB can account for nuances that even the human eye cannot notice, and as a result, there is redundancy in the colour values of the ochre streaks as some of the variations in streak colour are so subtle. Even so, there are noticeable differences in the colour streaks that the ochre pieces from HF produce. Streak colours in Quadrant 1 (mean LAB value = 51, 25, 36) are visibly darker and redder. Quadrant 2 (mean LAB value = 76, 9, 49) are lighter and appear orange or dark yellow. Quadrant 3 (mean LAB value = 85, 0, 32) are light yellow and beige, while Quadrant 4 (mean LAB value = 47, 18, 23) contain the darkest colour streaks and appear dark brown or dark purple-brown. Quadrant 1 and 4 contain the highest percentage of colour streaks (combined 57%), indicating that darker, redder and more purple hues are more common amongst the streaks produced by HF non-modified ochre artefacts.

### Anthropogenically modified ochres

**Fig 8:**
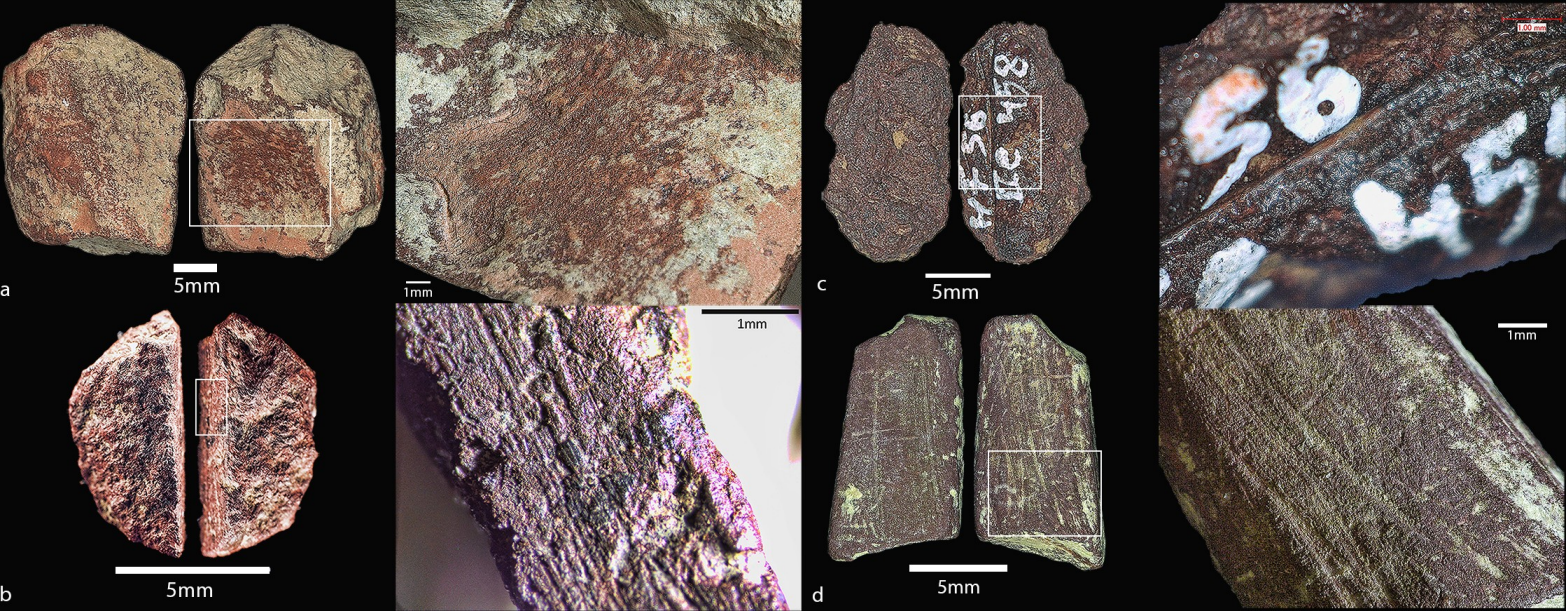
a-d. Anthropogenically modified ochre pieces from Hohle Fels. Artefact numbers and details are as follows: a) 59.IIa.629, ground out cavity, Magdalenian; b) 100.IIb.263, ground surface with striations, Gravettian; c) 56.IIb.458, scoring marks, Gravettian; d) 68.IIa.124, ground surfaces with resulting “crayon” shape, Magdalenian.

**Fig 9:**
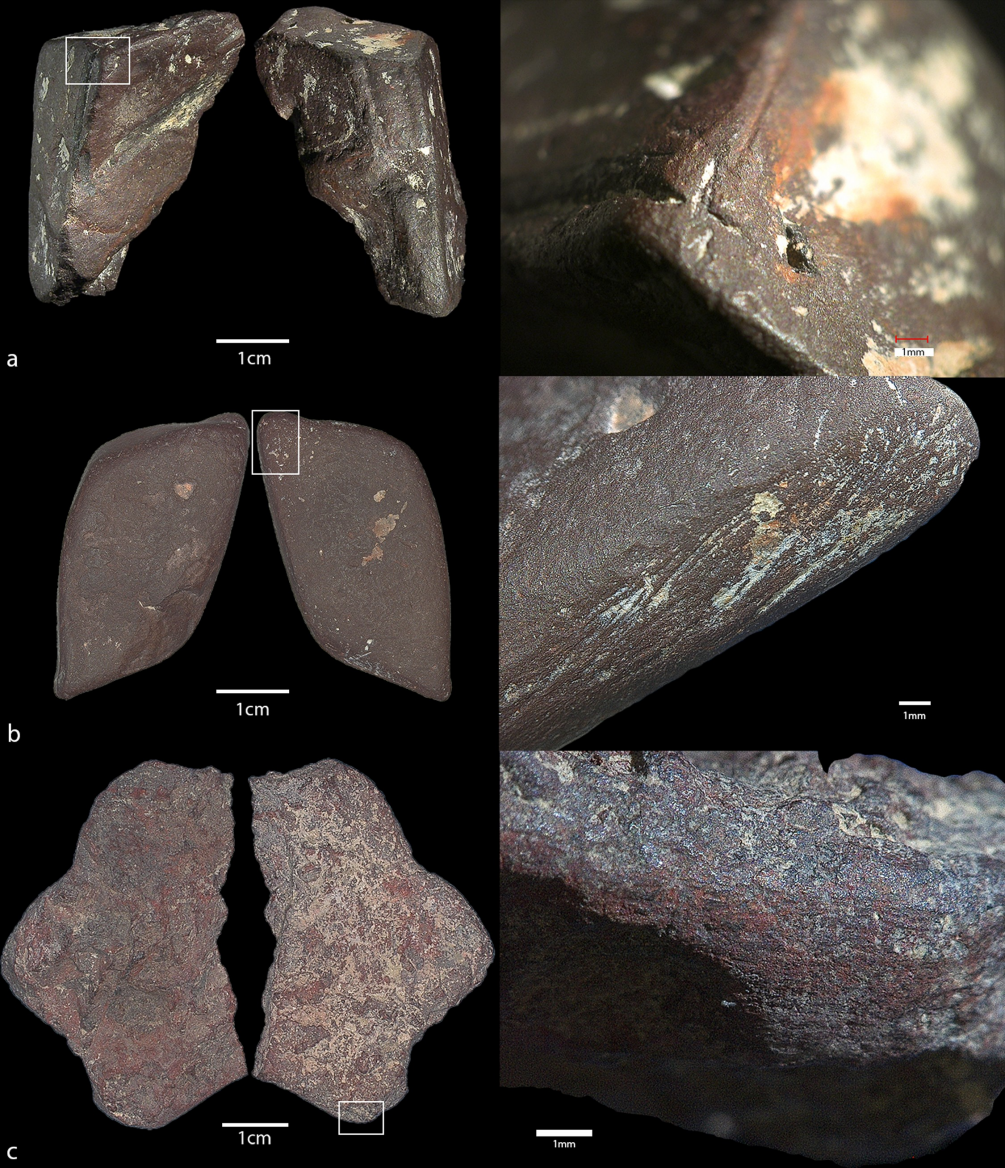
a-c: Anthropogenically modified ochre pieces from Hohle Fels. Artefact numbers and details are as follows: a) 102.Ib.420, scoring and grinding marks, Magdalenian; b) 102.Ia.399, ground and smoothed surfaces, Magdalenian; c) 49.IIa.218, ground surface with striations, Magdalenian.

**Fig 10:**
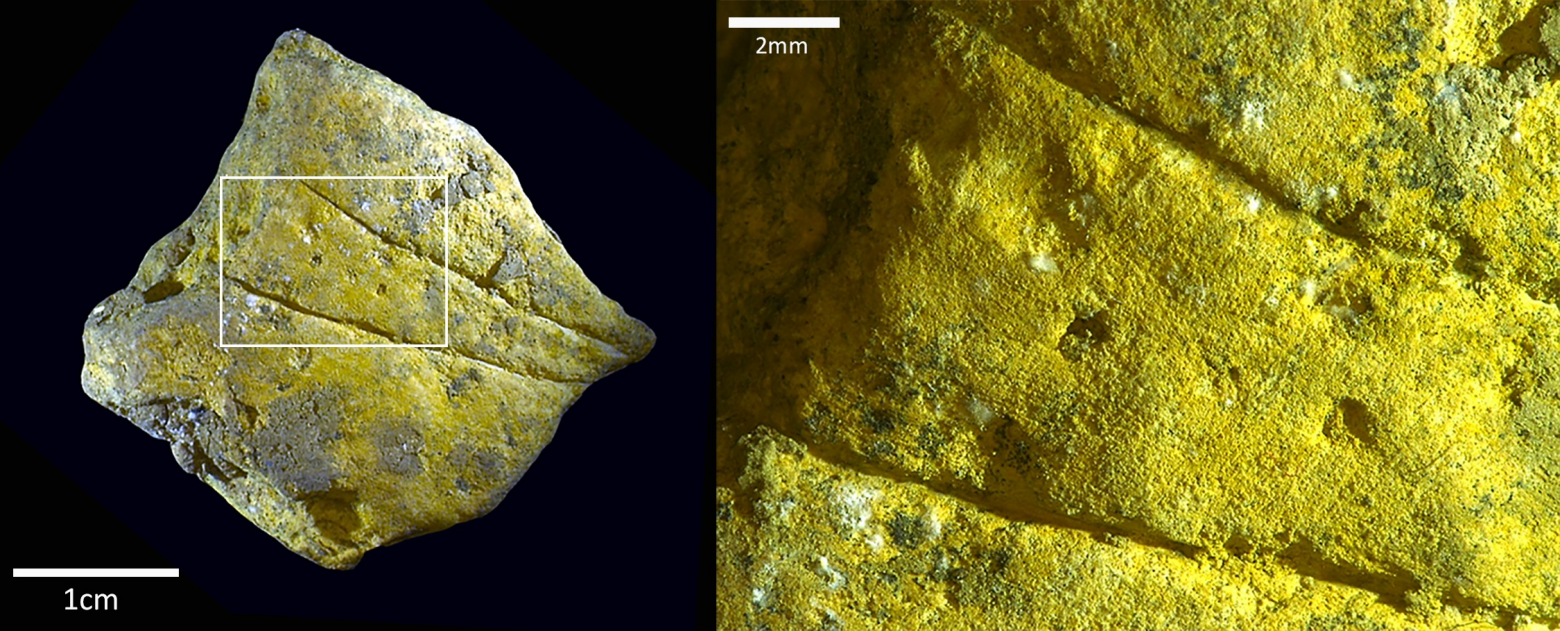
Modified ochre from the Hohle Fels Aurignacian. Artefact 87.1271, from the Aurignacian layer IIIa, showing two incisions with dots in the middle on a prepared surface.

Of the total HF ochre assemblage (n = 869), 3.1% (n = 27) bear traces of anthropogenic modification (images of selected modified pieces are shown in Figs 8 and 9). Due to the small assemblage size, our analysis of these artefacts is more qualitative as large-scale and long-term trends cannot be inferred. However, of the modified pieces, there is a preference towards hematite ochres (n = 20, 74%) with silty (n = 14, 52%) or clayey (n = 10, 37%) textures. Dark purple (n = 13, 48%) and dark red (n = 8, 30%) are the most frequent colour categories, and only 25% of the assemblage is specular in appearance (n = 7). Table 5 outlines the presence of different modification types present on the modified ochre artefacts, including grinding, rubbing, scoring, polish, and shaping (faceting and rounding). These traits often occur together, such as grinding leading to shaping and rubbing leading to polish. The combination categories included: *Shaped and rubbed*, *scored*, *rubbed and scored*, *rubbed*, *rounded*, *ground and rounded*, *ground*, and *faceted*. Overall, *ground and rubbed* and *rounded* are the most common modifications with five artefacts each, followed by *ground* then *rubbed and scored*. Of the artefacts with evidence of scoring (whether it was rubbed beforehand or not), only one seems to have incisions that possibly form an engraving or a design. This ochre artefact (Fig 10) is the only modified ochre from the Aurignacian (AH IIIa) and also the only piece of yellow ochre collected and included in this analysis due to the presence of the modifications. It shows two deep scored incisions on a prepared flattened surface. The incisions are deeper on the right side where they are closer together, then taper off both in width and in depth. The transitioning depths suggest a sort of “sweeping” motion with the incisions, which were likely made with a stone tool or sharpened bone artefact.

**Table 5:** Modifications types on Hohle Fels ochre. Anthropogenic modification categories for ochre artefacts from all time periods.

Period	Grinding	Rubbing	Scoring	Polish	Shaping
Holocene	1		1	1	1
Magdalenian	9	14		11	8
Gravettian	3	2	2	3	2
A/G trans		1		1	
Aurignacian		1	1		
**Total**	13	18	4	15	11

### Ochre-related artefacts (residues)

**Fig 11:**
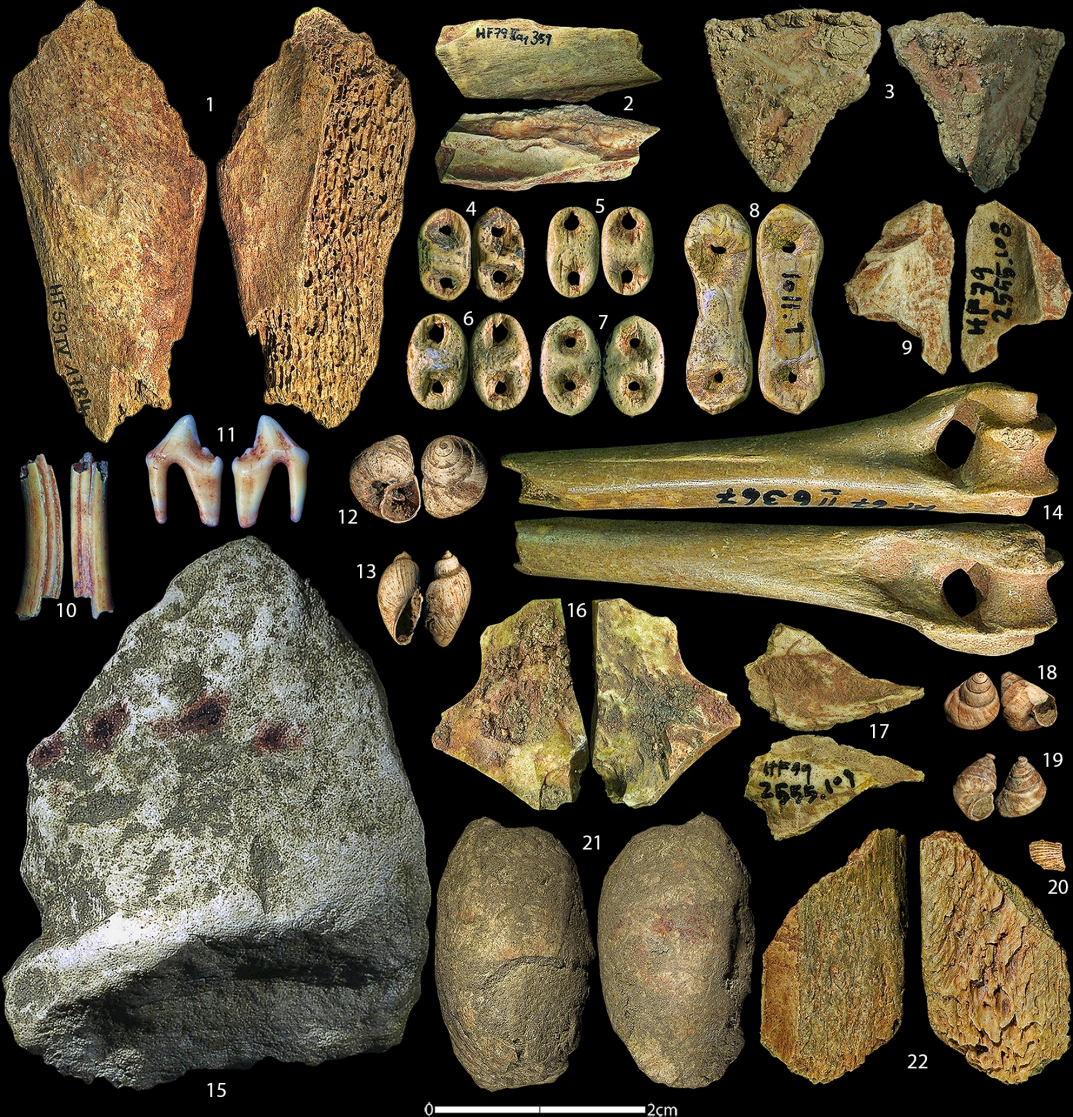
Ochre related artefacts from Hohle Fels. Assorted artefacts containing red ochre residues from all time periods at HF. Artefacts include faunal elements, teeth, mammoth ivory beads, freshwater snail shells, a fossilized mollusc, and lithics. Numbers correspond to Table D in S1 Table.

The ochre-related artefact category contains a variety of material types including lithics, limestone, bone, tooth, shell, mammoth ivory, and fossil (Fig 11). In total, 256 individual artefacts with traces of red residues were found and collected from the HF collection. A detailed overview of the artefact types with traces of red residues is shown in Table 6.

**Table 6:** Ochre related artefact types per time period from Hohle Fels. Distribution of material types per time period of artefacts with red colour residues, as well as type of residue and final decision as to the nature of the residue application. Abbreviations are as follows: A = Aurignacian, A/G = A/G transition, G = Gravettian, M = Magdalenian, H = Holocene.

		A n = 110	A/G n = 14	G n = 56	M n = 57	H n = 10	Total n = 247
**Material** **type**	Bone	7	4	3	18	1	**33**
	Limestone	79	10	29	12	5	**135**
	Fossil			1	5		**6**
	Ivory	20		4			**24**
	Shell			12	11	2	**25**
	Other stone	4		1	2		**7**
	Tooth			6	9	2	**17**
**Residue** **type**	Applicator	1		2			**3**
	Grindstone			1	1		**2**
	Per. ornament	19		11	7	2	**39**
	Painting				10	1	**11**
	Ochre mixture	5		1	1		**7**
	Container	2					**2**
	Total	27		15	19	3	**64**
**Final** **decision**	Direct application	19		9	10	2	**40**
	Indirect colouring	10	4	16	33	4	**67**
	Natural	81	10	31	14	4	**140**
	Total	110	14	56	57	10	**247**

Of the artefact types with coloured residues, the most frequent is limestone with 104 (41%) artefacts. The previously published seven limestone pieces with patterns of red dots [99, 100, 106, 110, 126, 127] are included in this total. Bone artefacts are the second most frequent category with 33 (13%) specimens, followed by mammoth ivory (n = 24, 9.4%) and shell (n = 23, 9%).

The nature of the residues on the artefacts was also classified if the residues were considered anthropogenic, whether intentional or secondary, in origin. These categories include *applicator*, *grindstone*, *personal ornament*, *painting*, *ochre mixture*, and *container*. The most frequent residue types are personal ornaments with red colouring on 39 (15.8%) (Fig 11.4–11.8) artefacts. Personal ornaments are counted as a residue type because mammoth ivory beads and tooth beads, specifically reindeer tooth, were found containing red residues. It is yet unestablished whether the origin of these residues was due to direct action or the result of indirect processes, such as the rubbing off of ochre onto the beads. The category *painting* includes the painted limestone pieces as well as artefacts with residues forming what appear to be a defined shape or outline, and total 11 artefacts (4%). A mollusc fossil from the Magdalenian with a red circle on one side is included in this category and is shown in Fig 11.21. *Ochre mixture* is the third most frequent (n = 7, 2.7%) and designates residues containing a thick visual application of ochre with small fragments of quartz and charcoal mixed in (such as the reindeer rib fragment shown in Fig 11.1).

**Fig 12:**
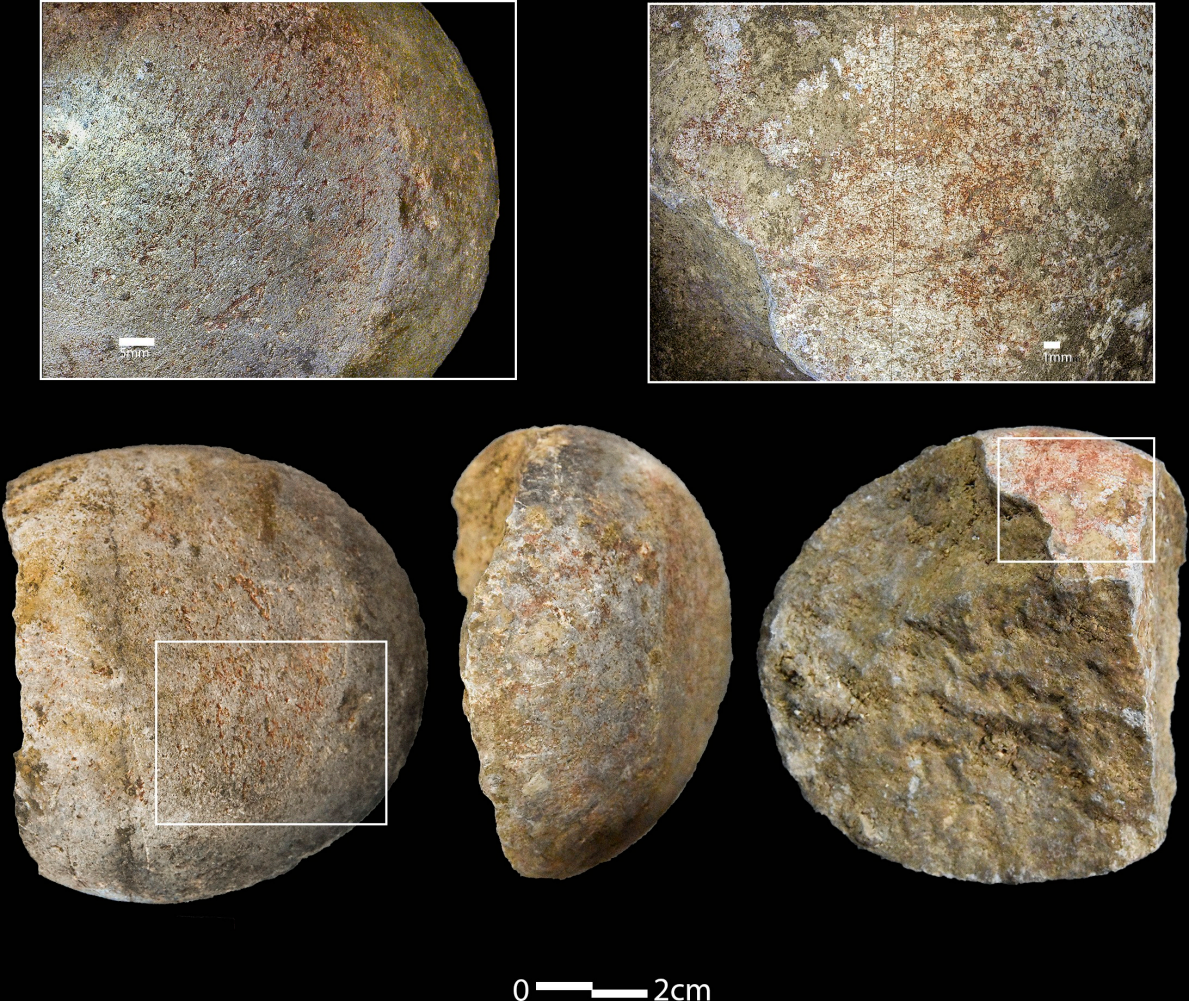
Ochre grindstone from the Hohle Fels Gravettian. Dolomitic limestone cobble which was likely used as a grindstone (#112.1157). Inserts show presence of ochre powder as well as striated surfaces.

One of the least common categories, *grindstone*, is one of the most important regarding ochre use at the site. One of the grindstones from HF, artefact #112.1157 (Conard and Malina, 2019, in prep) was found in 2018 and comes from the Gravettian layer IIb. It is a rounded fine-grained oolitic dolomite cobble with one faceted surface containing abrasive striations filled with red powder (Fig 12). It measures 7.8 x 7.1 x 4.1 cm and weighs 316 g. Red residues are also visible on a side surface of the artefact, which is also rounded and contains striations. Several notches and percussion marks on this side surface suggest the artefact’s use as a hammerstone in addition to a grindstone.

For the *residue origin* category, the majority of the artefacts contain red residues likely as a result of non-anthropogenic post-depositional processes, or *natural* processes (n = 140, 56.7%). This is followed by *indirect colouring* (n = 67, 27.1%), and lastly as a *direct application* or as a result of anthropogenic intent (n = 40, 16.2%). We note that the proposed residue types and final decisions are based solely on visual inspection. We also considered find context and sediment description in determining whether residues could have a natural or anthropogenic origin.

## Discussion

In the following section, we will first discuss the temporal changes in qualitative ochre characteristics throughout the sequence and how this is related to behavioural or environmental changes at HF during the late Pleistocene. We will further elaborate on the modified ochres as well as the ochre-related artefacts and how these reflect behavioural processes and what we can infer about symbolic processes surrounding the use of ochre pigments at the site. Following this, we will discuss the stratigraphic integrity of the site and the geogenic vs anthropogenic transportation of ochre materials to the site, as well as the role that post-depositional and anthropogenic processes likely played in the current physical condition of the ochre artefacts at HF. Lastly, we discuss the evidence of ochre artefacts and how these reflect the nature of ochre use at the site and in Europe during the UP.

### Ochre qualities and characteristics at Hohle Fels

The HF ochre assemblage totals 869 artefacts, and the observed diachronic changes show clear trends in the types of ochre that were collected and deposited at the site. For example, there is a marked difference in textural and colour preference in the Aurignacian compared to the later periods. CIELAB results of the streak tests show a higher degree of lighter hues during the Aurignacian (71 avg. lightness), with darker hues becoming more frequent during the Gravettian (56 avg. lightness) and Magdalenian (53 avg. lightness). The significance of the differences of each time period were assessed using a post-hoc multiple comparisons test for the L* value mean between each pair. The null hypothesis was rejected in every comparison with the Aurignacian except for the A/G transition (Table G in S1 Table), showing that the lightness values for the Aurignacian are statistically significantly different (p < 0.05) when compared to most of the other time periods. This observed change in streak colour records either a shift in resource availability or a shift in preferences for deeper redder and purple hues in the later time periods. Furthermore, whereas the majority of the Aurignacian ochre assemblage consists of fine-grained sandy ochres with a red colour, this changes during the A/G transition layers where more silty textures are present in the assemblage. This latter pattern of silty texture preference also continues into the Gravettian and Magdalenian at HF. The relative amount of micaceous hematite also increases towards the end of the Pleistocene, first occurring with relatively low frequency in the Aurignacian (n = 41, 11%), peaking in the Gravettian (n = 128, 46%), and then again slightly decreasing in the Magdalenian (n = 47, 29%). The preference for hematite-rich ochre (n = 20, 74%) with silty and clayey textures is reflected in the anthropogenically modified ochre assemblage as well, though micaceous hematite is not as frequently anthropogenically modified (n = 7, 35%).

There is more variability in ochre texture and rock types in the Aurignacian than in the later periods at HF, as evidenced by the representation of all eight rock type categories and no types occurring more frequently than 46% (the Gravettian has 67.5% hematite with six categories represented, Magdalenian has 70% and also six categories).This narrowing of textural and material types throughout time suggest that ochre preferences were being continuously refined at the site. Though preferences and changes in behavioural patterns could account for the shifts in ochre types brought to the site, a fluctuating landscape and palaeoenvironment may have played a role as well. During the Aurignacian, the Swabian Jura maintained a relatively stable warm and wet climate with minor cold fluctuations, which gradually changed to a much colder climate during the Gravettian and Magdalenian [77, 79, 128]. These shifts impacted the physical landscape surrounding the cave and the river valleys by decreasing terrestrial forest and allowing for major erosional phases inside and outside the cave during the Gravettian and Magdalenian [79]. These dramatic landscape changes may also have shifted or impacted the accessibility of particular ochre sources, or allowed the identification and exploitation of new ones.

The shift in resource gathering strategies is also present in the modes of lithic raw material acquisition. During the Aurignacian, local lithic resource areas were preferred as evidenced by the abundance of the local *Jurahornstein*, a type of chert, at HF [129, 130]. The Gravettian and Magdalenian lithic assemblages contain both local and non-local lithic raw materials, indicating changes in resource acquisition later in the Pleistocene [90, 129, 131]. This change in behavioural patterns reflected in lithic acquisition patterns, and material culture more generally, has also been suggested to be closely linked to processes related to the construction of social and individual identity [132]. Porr [133] has, for example, noted a shift in symbolic material culture forms, from a smaller and more varied ivory figurine tradition in the Aurignacian to a higher standardisation of figurative forms during the Gravettian. Porr [133] has suggested that the variability in artefact categories during the Aurignacian is higher than in other periods because Aurignacian social groups were comparatively smaller, more flexible and more conducive to individualised expressions of identity in material objects. The greater standardisation in Gravettian objects, which is visible all-over Central Europe [134], is indicative of larger, socially more cohesive and regionally inter-linked groups [135, 136].

In the HF ochre assemblage, there are differences in the Aurignacian ochre materials versus the Gravettian and Magdalenian. The differences in ochre types, ochre textures, and streak colours (signifying powder colour) may also reflect changes in behaviours, with more individual-based experiential collecting during the Aurignacian followed by a shift to “standardised” mass-collecting at specific source areas for certain ochre types in later periods. Though the specifics of ochre acquisition strategies and source locations are still unknown, the existing assemblage suggests that there were several different ochre types available to the cave inhabitants and that these were accessed throughout the UP with different behavioural intensities.

### Discussion of the modified ochre assemblage

Though the modified ochre assemblage is comparatively small (n = 27), 18 of these contain apparent striations, and half of the total assemblage (n = 14) bears evidence of grinding. Soft-surface grinding or rubbing is even more frequent (n = 17) while scoring or incising is the least common modification type with only four known examples. The majority of the modified ochres have silty or clayey textures, and the general trend of darker purple and red hues is consistent with other ochre studies which report a preference for fine-grained ochre with deeper red and purple hues in later time periods [23, 119, 121, 123, 137].

The types of modification present on the ochre artefacts suggest that the production of pigment in the form of ground powder was the primary objective for the inhabitants of the cave. Grinding and pulverising are the most effective ways of creating pigment powder from solid ochre pieces [112]. It is also possible that the pieces were first prepared by grinding and after that used for direct application onto a soft material such as skin, or the pieces were used as “crayons” on hard surfaces. The yellow ochre artefact from the Aurignacian (Fig 9) represents the only modified artefact with evidence for engraving or the application of a design. The two lines form a V shape and contain two dots between them. Aside from interpreting the design as figurative, possible scenarios could include “testing”, such as for lithic or bone artefacts for sharpness, or for the pigment or textural potential of the yellow ochre artefact. It is unlikely that this engraving was meant for pigment extraction as engraving or incising produces little to no pigment powder.

### Discussion of ochre-related artefacts

The ochre-related artefacts offer further insights into the way ochre was processed and used at HF. In cases where the anthropogenic application is certain (e.g. the painted limestone pieces), ochre was collected, processed, and lastly applied to these artefacts before deposition. They represent a direct link between physical artefact, intermediate state (powder) and intentional behaviour (rock painting). The presence of two ochre grindstones in the Gravettian and Magdalenian contexts at the site indicate that ochre was processed at least at a minimal level during these time periods. These provide a link between the operational chain of ochre use at HF, considering the presence of modified and unmodified ochre artefacts, a grindstone as an object to pulverise ochre into a usable pigment powder, and painted artefacts indicating its use as a pigment. Furthermore, the textural varieties of these grindstones could suggest a technological awareness of which grain sizes are more useful for grinding ochre artefacts into finer powders [120]. This entire sequence of ochre processing attests to the entrenchment of ochre into behavioural patterns of the cave inhabitants during the UP.

The personal ornaments with red residues also offer insight into the nature of ochre use at the site. During the Aurignacian, mammoth ivory was the preferred medium for creating personal ornaments as evidenced by the frequency of ivory beads during this period [5, 138, 139]. This changes during the Gravettian to an increase in faunal elements, such as teeth from cave bears, foxes and wolves [140, 141]. Regardless of the reasons for this change in material preferences, red residues are found on personal ornaments during all time periods and on ivory and other faunal ornaments.

There are several possible scenarios why red ochre may have been applied to ivory and faunal beads. In the case of ivory, White [142] suggests that red ochre, specifically in the form of hematite, assisted in the creation of the ivory beads by facilitating the smoothing and polishing of the ivory surfaces, as well as helping grind the perforations in the beads [143]. This interpretation would also explain the predominance of residues appearing only in the perforations of the ivory beads, as is the case with all 24 ivory bead artefacts. Another scenario that explains the presence of red residues in the perforations is that the string or other fabric that the beads were worn on was painted with red ochre. Further analyses on use-wear traces as well as residue analyses might allow for more detailed insights into these scenarios. The personal faunal ornaments with traces of red residues are all reindeer teeth incisors. Similar reindeer teeth artefacts are found at other archaeological sites from the Magdalenian [103, 105, 144, 145], though HF also contains six from the Gravettian. It is believed that the lower jaw of a reindeer was broken and the lower incisors were sawn off while remaining in the gums and were worn like a pendant [103]. The presence of red ochre on these teeth suggests a use as a decomposition deterrent (in order to preserve the gums for longer), for aesthetic purposes, or that red ochre was worn as body paint and rubbed off onto the pendants. These scenarios are not mutually exclusive, and red ochre may have been applied both to slow putrefaction as well as to make the pendants red. Considering the types of personal ornaments and other artefacts collectively, red ochre was not used exclusively on one material or artefact type. Instead, the evidence suggests that no single explanation can account for all occurrences of ochre in the UP record of HF. Red ochre seemingly had significance on a multitude of levels and was perceived and interacted with accordingly (see also [23, 113, 120, 121, 123]).

### Stratigraphic integrity and human transportation of ochre materials

Throughout the UP, there have been several periods of soil formation, cave sediment erosion and river fluctuation in the Swabian Jura and more specifically in the Ach valley [79]. Climatic transitions during the Würm interstadials and stadials resulted in a lack of stabilising vegetation which led to water erosion in the surrounding area and of cave sediments. These shifts in climate also contributed to major erosional phases within the cave after ca. 26 ka BP, resulting in truncated Gravettian and Magdalenian deposits [79, 82, 146]. Soil surface instability, as well as possible resource scarcity [147], appeared to have resulted in abandonment phases in HF around 27–26 ka BP. Magdalenian groups migrated back into the area around 16.5–15.5 ka BP [91, 148] during a cool interstadial period, though these sediments were also eroded on a smaller scale. Erosion ceased ca. 12.5 ka BP and the remaining Magdalenian deposits were capped by a *Bergkies* accumulation [79].

The beginning of the Aurignacian at HF is characterised by an initially mild, dry phase followed by a warm wet phase, with the environment being largely tundra-based with boreal elements indicated by the presence of several characteristic pine species represented in the palynological record [149]. Micromorphological investigations show that there was little erosion of sediments into or out of the cave during this time period [77]. Furthermore, the presence of ochre artefacts in layers that are dense with lithic artefacts and bone debitage (AH Va) strongly correlate with periods of human occupation. We argue that this lack of erosion shows that the ochre materials of the Aurignacian were intentionally collected and brought to the site, rather than being deposited by landscape erosional processes.

However, the Gravettian and Magdalenian periods differ from the Aurignacian layers. The Gravettian began within a cold and wet climatic phase, which shifted towards increasingly cooler and drier conditions and was largely dominated by a tundra environment [149]. These fluctuations caused a decrease in arboreal and woody plant coverage leading to a higher rate of soil erosion due to increased water transportation in the landscape [77, 79, 149]. A higher rate of sediment movement, combined with more frequent periods of freezing and thawing, likely affected ochre materials within the cave. The non-eroded Gravettian deposits remaining in the cave yielded 278 ochre artefacts or approximately 32% of the entire assemblage. Comparatively, the Aurignacian contains ca. 43% of the entire assemblage, and the majority of these layers are considered as intact sediments. Though the Gravettian occupation at HF was not only short but also heavily eroded, the high amount of ochre artefacts indicate that the collection and use of ochre was a well-established by at least 29 ka BP.

Furthermore, the decrease in ochre rock type variability and the focus on mica-rich fine-grained hematite pieces producing darker and redder streaks suggest that specific source zones were known, sought, and continually accessed during the Gravettian and Magdalenian. This type of activity requires intimate knowledge of the landscape and the available outcrops, and may thus indicate the development of more pronounced social and cultural preference for ochre textures, colours, and appearances. It may also suggest adaptability in a changing landscape (one ochre source eroded away, another one sought after and accessed), an increased movement range in order to access specific sources, or increased communication and trade with other social groups.

### On the nature and size of the assemblage

The size of the HF ochre assemblage (n = 869), and the highly fragmentary condition of the ochre artefacts themselves (average weight: 1.07 g) begs the question: why are there so few ochre pieces and why are they so small? Does the nature and condition of the HF ochre assemblage suggest that the people occupying HF during the UP only exhibited a limited range of ochre behaviours that were mainly opportunistic? In order to investigate this hypothesis and the range of geogenic processes occurring at the cave in the late Pleistocene, we propose four scenarios that may account for the assemblage size as well as the conditions of the artefacts:

1. *Ochre-use efficiency*. Experimental studies on ochre powder production show one of the most effective ways to produce fine-grained pigment is by pulverising the ochre using a mortar and pestle method [37, 123]. This technique is employed by modern-day indigenous groups in Africa, e.g. the Ovahimba in Namibia [41, 150], and it results in a solid piece of ochre being transformed entirely into powder. This type of ochre processing would thus leave little or no archaeological footprint, as experiments show that this form of processing leaves a barely discernible “splash pattern” of ochre at the processing site. Consequently, the prehistoric inhabitants of HF may have been very efficient in processing raw ochre nodules into a useable powder suitable for pigments, leaving only a few macroscopic (ca. 5 mm) ochre pieces.
2. *Post-depositional deterioration*. The extent and intensity of certain geogenic processes at HF, specifically erosion, freezing and thawing, contributed to the fragmentation and complete relocation of ochre artefacts contained within the cave [77, 79]. Thus, it is conceivable that the recovered HF ochre assemblage is only a partial representation of the ochres that were originally used and deposited, and that these were likely eroded out of the cave during the Gravettian and Magdalenian. This has further been evidenced by the recovery of a reworked bone fragment from a core sample at a depth of 8.2 m in front of HF, dated to the Gravettian (28,000 ^14^C BP), indicating that it was likely eroded out from HF [79]. The ochre artefacts remaining in the cave were situated in a moist and mobile environment with typical taphonomic processes taking place, such as the phosphatisation of sediments, cryoturbation, and trampling from animals or other cave visitors, to name a few. These processes are detrimental to ochres, especially for the fragile clay-based iron oxides that are abundant in the region, and may most certainly have contributed to the degradation of the ochre artefacts within the cave. Furthermore, the processing of sediments and artefacts post-excavation, such as water-screening, likely contributing to the eradication of certain modification traces as evidenced by experiments on the secondary processing of ochres by other researchers [35].
3. *Limited ochre use*. Lastly, we consider the possibility that ochre use was simply not as prevalent at HF as at other sites during the late Pleistocene. One may also argue that relatively few artefacts with definite traces of direct anthropogenic ochre pigment application, namely the painted limestone pieces (n = 7), is further evidence that UP occupants at HF did not regularly interact with ochre. This scenario is also potentially impacted by the availability of ochre in the landscape. Though sources of ochre are present in the modern-day Swabian Jura landscape (see [69]), it is possible that these were not known or accessible during the UP. Restricted access to sources would in turn limit the availability and subsequent use of ochre at HF.
4. *Landscape mobility/seasonal migration*. Archaeological evidence shows that HF was likely an occupation site during certain seasons, but it is probable that there were several temporary open-air sites corresponding to seasonal hunting and foraging cycles. These landscape mobility patterns, also seen with indigenous groups in Africa [151, 152], could potentially split up certain types of behaviour, such as ochre use, amongst the different camps and temporary habitation sites. Lithic raw material studies show that the total area covered by Aurignacian populations was ca. 7,000 km^2^, and the Gravettian may have been up to ca. 9,000 km^2^ [153]. Based on these possible landscape movements and migrations, ochre may have been collected and processed elsewhere, and the deposited assemblage at HF may only represent a fraction of the total amount of utilized ochre.

The average size (9.78 mm) of the individual ochre artefacts and the condition of the ochre assemblage is likely due to a combination of the first two scenarios, and may have been further compounded by seasonal migrations represented by the last scenario. Given the post-depositional influences outlined in the second scenario, it is likely that these had at the very least a minor effect on the assemblage size and artefact size. This is further supported by modern-day experiments, which show that artefact cleaning and processing can almost wholly destroy a soft clay-based ochre artefact even after it is excavated [35]. It is plausible that the existing assemblage is a minimum estimate of the total size, and the original assemblage was most likely much more substantial. Furthermore, few informative open-air sites in the Swabian Jura have been identified [1, 154]. This is due in part to the intensity of landscape and environmental changes which had impacted these sites on a greater scale than the cave sites, where entire occupational horizons have been eroded. Nevertheless, the interpretation of characteristic faunal species identified key periods of cave occupation during cold phases, indicating some seasonal flucutation of cave inhabitants in the Swabian Jura [155, 156].

Regarding the size of the total HF ochre assemblage, the number 869 can at first appear small, especially when compared to ochre assemblages from Africa that can total in the thousands [23, 33, 113, 120, 123]. However, we argue that this comparison is unwarranted, considering both the geographical and temporal spread of the ochre assemblages. For European contexts, the majority of ochre assemblages that have been reported in detail date to the Châtelperronian [49, 63, 66, 157] or the Acheulian [47]. At *Grotte du Renne* in France, which is perhaps the most extensively reported ochre assemblage in Europe, albeit 40 years after the end of the excavations, over 2,000 artefacts are reported from the Châtelperronian layers and include red, yellow and black colourants, weighing in total around 18 kg [49]. As for the UP, currently the only available comparison comes from an open-air Aurignacian site, *Régismont-le-Haut*, where 1,001 artefacts are reported, including both red and yellow ochre, weighing in total 730 g (compared to HF with 925.768 g) and 98% of the assemblage containing artefacts smaller than 2 cm [48]. Because the availability of published systematic overviews of ochre assemblages is limited in Europe for UP sequences, we cannot claim that the 869 HF ochre artefacts are or are not indicative of a limited ochre use scenario. Furthermore, our focus rested on red ochre artefacts, which do not occur naturally inside of the cave, and did not include yellow or black colourants which would have resulted in a dramatically different number (see Materials and Methods regarding yellow ochre at HF). The assemblage does, however, represent regular and long-term ochre use at an UP cave site in Central Europe, and can serve as a benchmark for comparisons of other ochre assemblages from European UP sites in the future. Further analyses, surveys for local and non-local ochre sources and future excavations may shed more light on ochre behaviours at the site.

Lastly, we consider the artefacts with traces of red residues as indicators of ochre use at the site. The appearance of residues on several artefacts types, including bone, ivory, stone, and shell, while similar artefacts from similar contexts have no colourants, suggests that ochre was processed in the cave in specific areas at different points in time. Furthermore, the presence of two grindstones suggests that ochre was processed at the site during the Gravettian and Magdalenian. If the colouring was the result of post-depositional processes, the appearance would likely be more random and would cover artefacts from similar and possibly neighbouring contexts. If the colouring was caused by staining from the overlying sediment, we would also expect this to be visible on associated artefacts, especially artefacts with porous textures such as bone or ivory, and this is not the case. It is possible that these residues were not intentionally applied and the artefacts were merely “innocent bystanders” during different ochre processing phases; however, this is still indicative of ochre behaviours at the site. It is our future goal to conduct more intensive analytical investigations on artefacts with colour residues in order to identify the elemental constituents of these residues, and perhaps extend and enhance our knowledge on the range of ochre behaviours at HF.

### Ochre behaviour and ochre use at Hohle Fels cave during the Upper Palaeolithic

Ochre use was already well entrenched in the behavioural repertoire of AMHs by at least 80 ka BP [23, 32, 33, 113, 114, 122, 158] in Africa and the Levant. Though there is a sizeable contextual separation in both time and space between African and European Pleistocene populations, many of the behaviours observed at these sites are shared, such as the creation of stone tools, personal ornaments, and ochre and pigment use. Furthermore, the body of evidence showing pigment and ochre use by European Neanderthals is continuously growing [58, 59, 62, 64, 66, 67], which further supports the hypothesis that ochre use was well in place in Europe even before the onset of the UP. Whether these behaviours migrated with hominin populations as they traversed into regions outside of Africa, or whether they were independently developed, is still uncertain. However, working towards understanding the extent and range of ochre behaviours in Europe and comparing these assemblages globally will enhance our knowledge of ochre materials and how related behaviours evolved over time and across space.

At HF, red ochre artefacts occur in all stratigraphic contexts at the site. Red ochre is highly present during the UP time periods and is found alongside an assortment of other artefacts that contain red residues, as well as artefacts that were directly painted with ochre pigments. The different lines of evidence show that ochre was recognised in the landscape, collected, transported, and brought to the site and subsequently processed in order to create pigment powder. This pigment powder was then mixed with a suitable binder and used to create painted dots on a series of limestone fragments, and was likely directly or indirectly applied to other items such as ivory and tooth beads. This series of actions, recognition, collection, transportation, modification, application, and deposition represent part of the operational chain or *chaîne opértoire* of ochre use at HF. Among other aspects, these actions and cognitive processes and foci are connected to the perception of need [159], a procedural knowledge [160], a preference for specific textures of colours, and a desire to utilise and thus “create”. The presence of ochre throughout the UP suggests that the recognition of ochre as an item worth collecting was already well represented in the behavioural repertoire of populations living in the Swabian Jura during the earliest phases of modern human occupation in Europe. Whether or not this behaviour was transported from other regions, such as Africa or the Levant, is as of yet uncertain. The total range of behaviours surrounding ochre use is also unknown. Several theories propose the use of ochre for aesthetic purposes or in order to alter or enhance physical appearances [161], as a form of sham-menstruation by females in order to secure resources during vulnerable times [20, 25, 28], or for a variety of other more utilitarian purposes such as a hafting adhesive or sunscreen [35, 39–41].

We believe the most likely scenario is that both functional and ritual uses were occurring simultaneously and perhaps intertwined together, as is often the case in ethnographic examples regarding ochre use [14, 150, 162]. Our goal is instead to explore and expand the current knowledge base of ochre use during the earliest modern human cultural phases in Europe. The ochre assemblage of 869 individual artefacts, as well as the artefacts with traces of red residues, suggest that these behavioural traits were well in place by the arrival of AMHs in Europe and were intricately woven into the complex cultural-symbolic framework already shown in other forms of material culture, such as personal ornaments and figurines. Our analysis has also demonstrated that pigment selection, acquisition and use show significant patterns of change and stability over time. These processes can be linked to changes in the environment and respective social behaviours and the related dynamics of identity and group formation. Thus, the use of pigment and ochre artefacts at Palaeolithic cave sites is considered to be a further thread in the fabric of behaviours of the earliest modern human populations in southern Germany and Europe more generally.

## Conclusion

In this paper, we have presented the first systematic assessment of the HF ochre assemblage dating to the UP, and the first analysis of a diachronic sequence of ochre artefacts from the UP in Central Europe. Through qualitative analysis of the nearly 900 ochre pieces, we have demonstrated that UP populations occupying the cave collected, transported, and used ochre regularly throughout the Aurignacian, Gravettian, and Magdalenian periods. They ground ochre to produce pigment powder, and they used these pigments to paint geometric motifs on rock slabs and possibly to colour other artefacts, such as personal ornaments and faunal remains. Consequently, we suggest that ochre played a significant role in practical and symbolic aspects of the behavioural repertoire of UP groups at HF and that these behaviours were transferred and shared throughout the millennia. We believe the qualities presented here and their evolution over time do coincide with those observed in other MSA sequences. In Europe, the few existing reports on ochre studies in UP contexts do not allow us to make robust regional comparisons; though with combining the existing research, we can ascertain that ochre behaviours were already in place by at least the Aurignacian in Europe [48, 49, 63]. While only a few ochre pieces (n = 27) show evident traces of anthropogenic modification, we relate the fragmentary state of the assemblage primarily to local preservation conditions and to the nature of ochre powder production (grinding and pulverising). The original ochre assemblage deposited at HF may thus have been considerably more substantial, and the documented ochre assemblage should be regarded as a minimum representation.

This study demonstrates that despite poor preservation conditions and a fragmentary record, important observations and inferences are still attainable. Thus, we hope that this study may serve as a starting point for future research, from which regional European comparisons can more robustly be made. That may ultimately expand our understanding of ochre use during the UP and how our human ancestors interacted with and were influenced by this fascinating material.

## Supporting information

S1 Fig **A-C. Previously reported ochre and ochre-related finds from Hohle Fels cave**.
**Fig A: Modified ochre pieces from Hohle Fels.** Previously found Hohle Fels ochre artefacts: a) Specular hematite piece with two facets, #102.630.1; b) Red chalk “crayon” piece with four striated surfaces, #102.555.1 (from [1]) (photos by E. Velliky); c) Rounded Rondelle-shaped fragment, #110.434.1; d) Two refitted Rondelle artefacts from hematite, #110.1104.1 & #110.992 (from [2]) (photos by M. Malina, 2009).
**Fig B: Painted limestone fragments from the Magdalenian of Hohle Fels.** Find numbers: a) limestone, #44.92 (from [3]) (photo by M. Malina); b) limestone, #110.985, c) limestone, #135.197 (from [2]) (photos by M. Malina); d), limestone, #55.253 (from [4]) (photo by H. Jensen); e) dolomitic limestone, #102.487, f) dolomitic limestone, #102.495 (from [1]) (photos by M. Malina); g) dolomite, 67.?? (from [5]) (photo by H. Jensen).
**Fig C: Previously found faunal elements with traces of red residues from Hohle Fels.** Descriptions and find numbers: a) broken long bone shaft, #14.69, Magdalenian (photo by H. Jensen), b) reindeer cranial fragment, #89.48, Magdalenian, c) cave bear temporal fragment, #29.1484.14, Aurignacian (photos by A. Blanco-Lapaz).
(PDF)

S1 Table **A-J. Artefact numbers, contextual details, and statistical tests for corresponding figures**.
**Table A: Fig 3 (Hohle Fels Aurignacian) artefact descriptions**.
**Table B: Fig 4 (Hohle Fels Gravettian) artefact descriptions**.
**Table C: Fig 5 (Hohle Fels Magdalenian) artefact descriptions**.
**Table D: Fig 10 (Hohle Fels artefacts with residues) artefact descriptions**.
Table E: Means and standard deviations for L*A*B* values per time period.
Table F: One-way ANOVA results for measured L* values for each time period.
Table G: Tukey-Kramer post-hoc test data for L* (lightness) colour values on Hohle Fels colour streaks per time period.
**Table H: Hohle Fels ochre rock type sorted by archaeological horizon (AH) and time period.** Abbreviations are as follows: H = Holocene, M = Magdalenian, G = Gravettian, A/G = A/G transition, A = Aurignacian. For the rock types: RS = Red sediment, DL = Degraded Limestone.
**Table I: Hohle Fels ochre textures sorted by archaeological horizon (AH) and time period.** Abbreviations are as follows: H = Holocene, M = Magdalenian, G = Gravettian, A/G = A/G transition, A = Aurignacian. For the textures: Med. Sand = Medium grained sand.
**Table J: Hohle Fels ochre colours sorted by archaeological horizon (AH) and time period.** Abbreviations are as follows: H = Holocene, M = Magdalenian, G = Gravettian, A/G = A/G transition, A = Aurignacian. For the colours: BR = Brick Red, DP = Dark Purple, DR = Dark Red, LP = Light Purple, LR = Light Red.
(PDF)
