# Supplementary material for: Ochre and pigment use at Hohle Fels cave: Results of the first systematic review of ochre and ochre-related artefacts from the Upper Palaeolithic in Germany
Source: PLoS One. 2018 Dec 27;13(12):e0209874. doi: 10.1371/journal.pone.0209874 (PMC6307870; doi:10.1371/journal.pone.0209874)
Supplement: S1 Fig — A-C. Previously reported ochre and ochre-related finds from Hohle Fels cave. Fig A: Modified ochre pieces from Hohle Fels. Previously found Hohle Fels ochre artefacts: a) Specular hematite piece with two facets, #102.630.1; b) Red chalk “crayon” piece with four striated surfaces, #102.555.1 (from [1]) (photos by E. Velliky); c) Rounded Rondelle-shaped fragment, #110.434.1; d) Two refitted Rondelle artefacts from hematite, #110.1104.1 & #110.992 (from [2]) (photos by M. Malina, 2009). Fig B: Painted limestone fragments from the Magdalenian of Hohle Fels. Find numbers: a) limestone, #44.92 (from [3]) (photo by M. Malina); b) limestone, #110.985, c) limestone, #135.197 (from [2]) (photos by M. Malina); d), limestone, #55.253 (from [4]) (photo by H. Jensen); e) dolomitic limestone, #102.487, f) dolomitic limestone, #102.495 (from [1]) (photos by M. Malina); g) dolomite, 67.?? (from [5]) (photo by H. Jensen). Fig C: Previously found faunal elements with traces of red residues from Hohle Fels. Descriptions and find numbers: a) broken long bone shaft, #14.69, Magdalenian (photo by H. Jensen), b) reindeer cranial fragment, #89.48, Magdalenian, c) cave bear temporal fragment, #29.1484.14, Aurignacian (photos by A. Blanco-Lapaz). (PDF) [file pone.0209874.s001.pdf]

Ochre and pigment use at Hohle Fels cave: Results of the first systematic review of ochre and ochre-related artefacts from the Upper Palaeolithic in Germany

Elizabeth C. Velliky*, Martin Porr, Nicholas J. Conard

* Corresponding author

E-mail: elizabeth.velliky@research.uwa.edu.au

S1 Figs A-C. Previously reported ochre and ochre-related finds from Hohle Fels cave.

Fig A: Modified ochre pieces from Hohle Fels. Previously found Hohle Fels ochre artefacts: a) Specular hematite piece with two facets, #102.630.1; b) Red chalk "crayon" piece with four striated surfaces, #102.555.1 (from [1]) (photos by E. Velliky); c) Rounded Rondelle-shaped fragment, #110.434.1; d) Two refitted Rondelle artefacts from hematite, #110.1104.1 & #110.992 (from [2]) (photos by M. Malina, 2009). 2

Fig B: Painted limestone fragments from the Magdalenian of Hohle Fels. Find numbers: a) limestone, #44.92 (from [3]) (photo by M. Malina); b) limestone, #110.985, c) limestone, #135.197 (from [2]) (photos by M. Malina); d), limestone, #55.253 (from [4]) (photo by H. Jensen); e) dolomitic limestone, #102.487, f) dolomitic limestone, #102.495 (from [1]) (photos by M. Malina); g) dolomite, 67.?? (from [5]) (photo by H. Jensen)............................ 3

Fig C: Previously found faunal elements with traces of red residues from Hohle Fels. Descriptions and find numbers: a) broken long bone shaft, #14.69, Magdalenian (photo by H. Jensen), b) reindeer cranial fragment, #89.48, Magdalenian, c) cave bear temporal fragment, #29.1484.14, Aurignacian (photos by A. Blanco-Lapaz). 4

Fig A: Modified ochre pieces from Hohle Fels. Previously found Hohle Fels ochre artefacts: a) Specular hematite piece with two facets, #102.630.1; b) Red chalk “crayon” piece with four striated surfaces, #102.555.1 (from [1]) (photos by E. Velliky); c) Rounded *Rondelle*-shaped fragment, #110.434.1; d) Two refitted *Rondelle* artefacts from hematite, #110.1104.1 & #110.992 (from [2]) (photos by M. Malina, 2009).

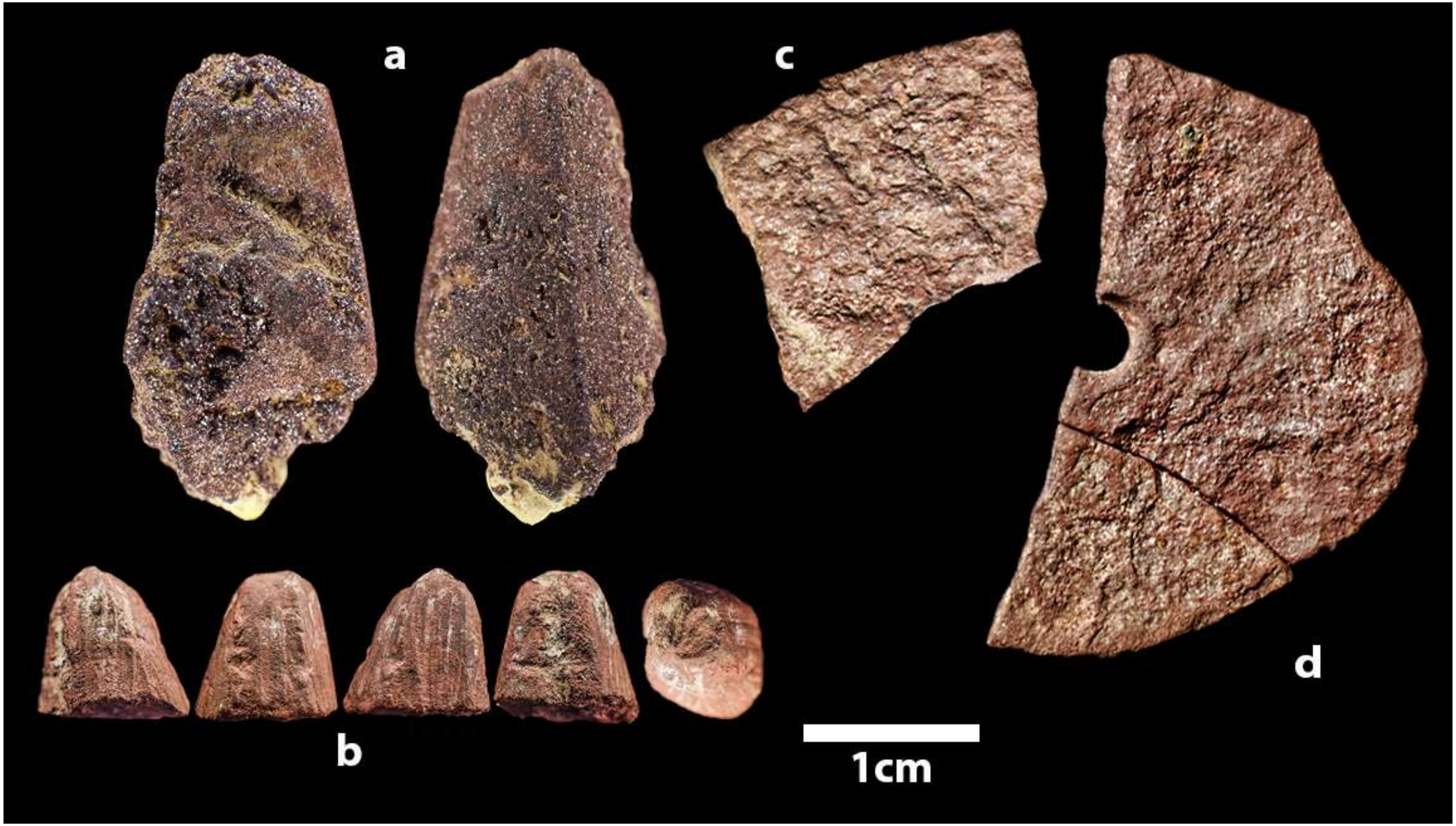

Fig B: Painted limestone fragments from the Magdalenian of Hohle Fels. Find numbers: a) limestone, #44.92 (from [3]) (photo by M. Malina); b) limestone, #110.985, c) limestone, #135.197 (from [2]) (photos by M. Malina); d), limestone, #55.253 (from [4]) (photo by H. Jensen); e) dolomitic limestone, #102.487, f) dolomitic limestone, #102.495 (from [1]) (photos by M. Malina); g) dolomite, 67.?? (from [5]) (photo by H. Jensen).

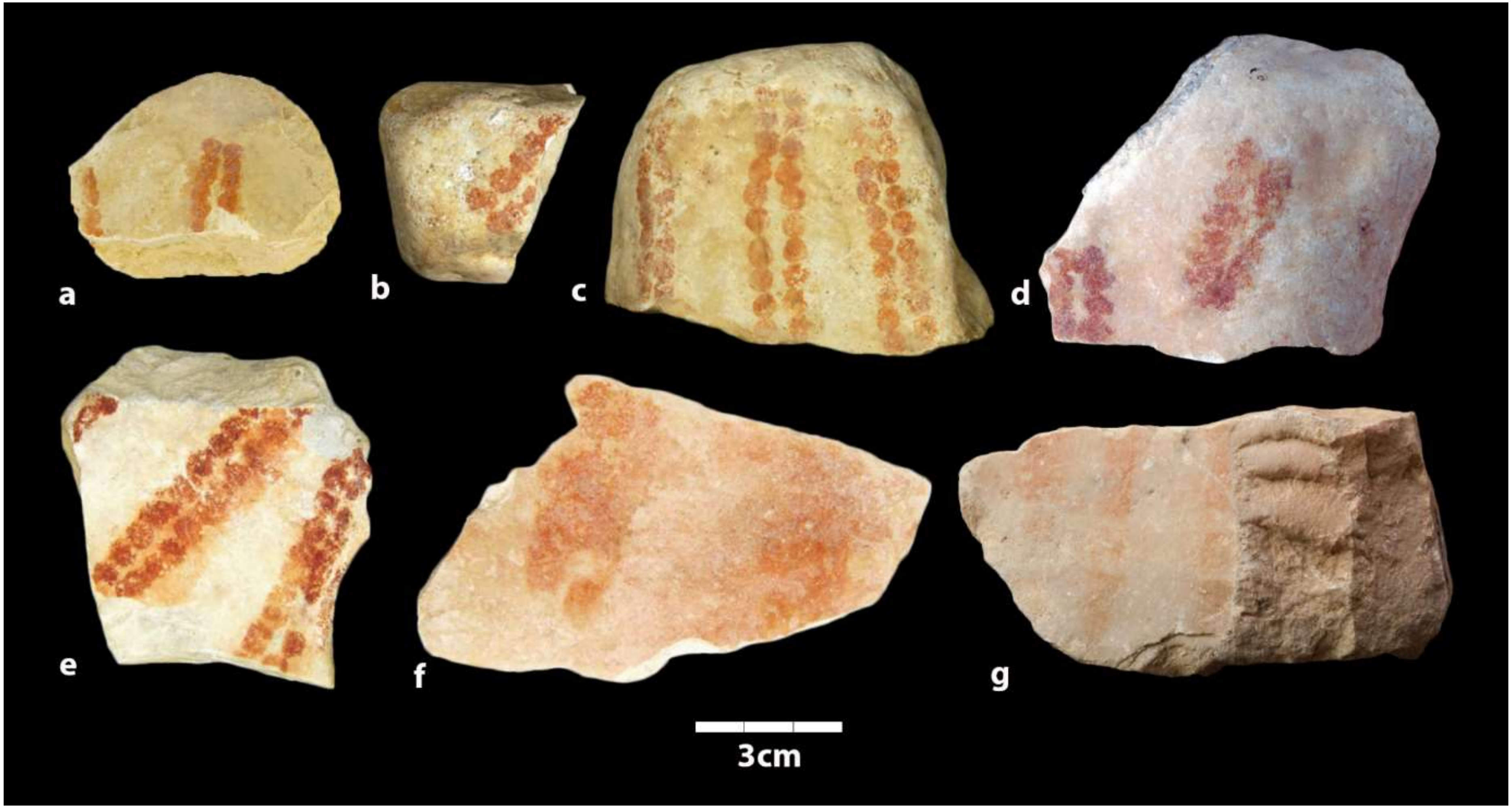

Fig C: Previously found faunal elements with traces of red residues from Hohle Fels. Descriptions and find numbers: a) broken long bone shaft, #14.69, Magdalenian (photo by H. Jensen), b) reindeer cranial fragment, #89.48, Magdalenian, c) cave bear temporal fragment, #29.1484.14, Aurignacian (photos by A. Blanco-Lapaz).

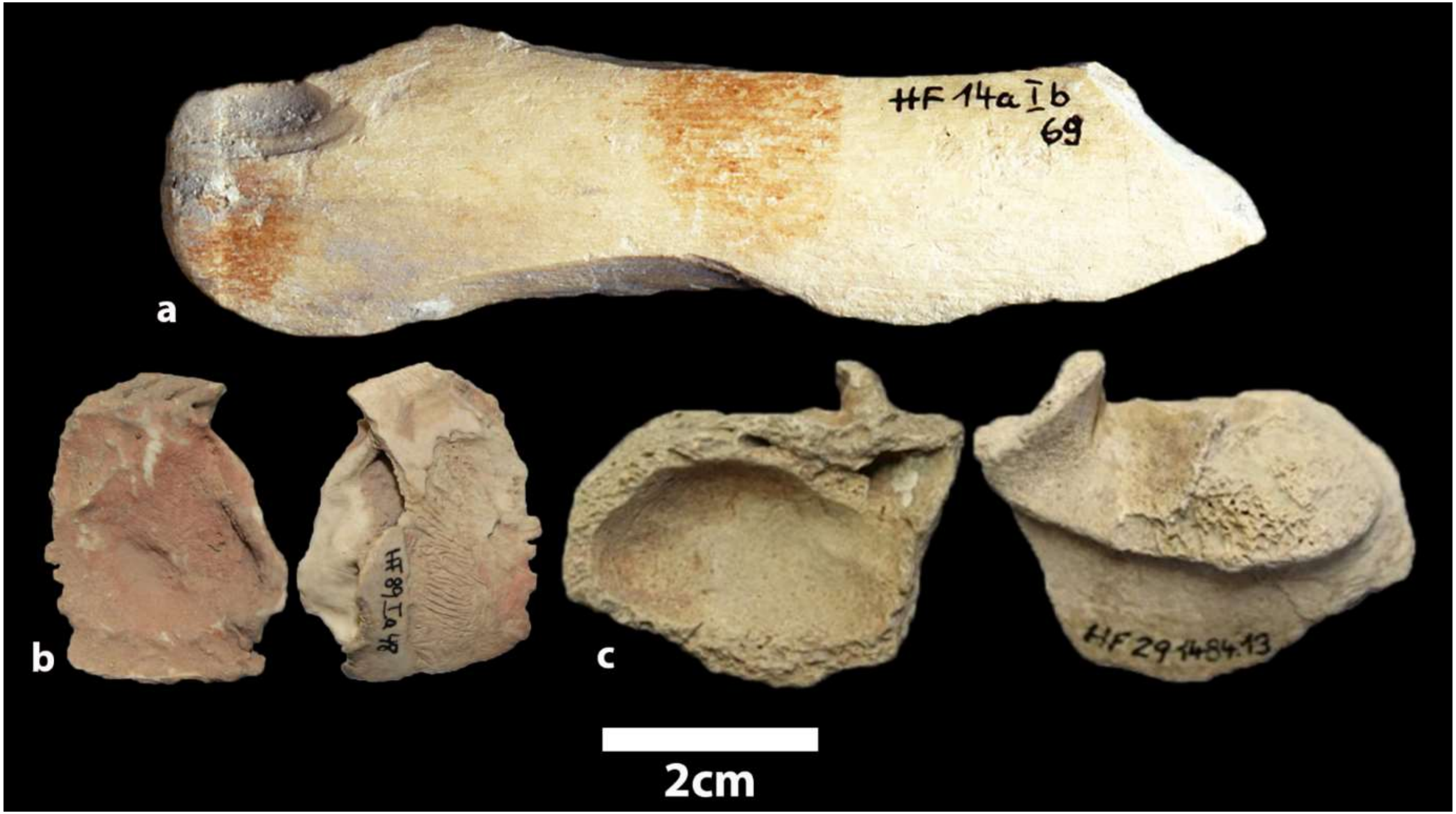

References

1. Conard NJ, Malina M. Neue Belege für Malerei aus dem Magdalénien vom Hohle Fels. Archäologische Ausgrabungen in Baden-Württemberg 2009. Stuttgart: Theiss; 2010. p. 52-6.
2. Conard NJ, Malina M. Neue Eiszeitkunst und weitere Erkenntnisse über das Magdalénien vom Hohle Fels bei Schelklingen. Archäologische Ausgrabungen in Baden-Württemberg 2010. Stuttgart: Theiss; 2011. p. 56-60.
3. Conard NJ, Malina M. Vielfältige Funde aus dem Aurigancien und ein bemalter Stein aus dem Magdalénien vom Hohle Fels bei Schelklingen. Archäologische Ausgrabungen in Baden-Württemberg 2013. Stuttgart: Theiss; 2014. p. 58-63.
4. Conard NJ, Uerpmann H-P. Die Grabungen 1997 und 1998 im Hohle Fels bei Schelklingen, Alb-Donau Kreis. Archäologische Ausgrabungen in Baden-Würrttemberg 1998. Stuttgart1999. p. 47-52.
5. Scheer A. Neue jungpaläolithische Funde aus dem Hohle Fels bei Schelklingen Alb-Donau-Kreis. Archäologische Ausgrabungen in Baden-Württemberg. 1994:24-7.
