# Supplementary material for: Ochre and pigment use at Hohle Fels cave: Results of the first systematic review of ochre and ochre-related artefacts from the Upper Palaeolithic in Germany
Source: PLoS One. 2018 Dec 27;13(12):e0209874. doi: 10.1371/journal.pone.0209874 (PMC6307870; doi:10.1371/journal.pone.0209874)
Supplement: S1 Table — A-J. Artefact numbers, contextual details, and statistical tests for corresponding figures. Table A: Fig 3 (Hohle Fels Aurignacian) artefact descriptions. Table B: Fig 4 (Hohle Fels Gravettian) artefact descriptions. Table C: Fig 5 (Hohle Fels Magdalenian) artefact descriptions. Table D: Fig 10 (Hohle Fels artefacts with residues) artefact descriptions. Table E: Means and standard deviations for L*A*B* values per time period. Table F: One-way ANOVA results for measured L* values for each time period. Table G: Tukey-Kramer post-hoc test data for L* (lightness) colour values on Hohle Fels colour streaks per time period. Table H: Hohle Fels ochre rock type sorted by archaeological horizon (AH) and time period. Abbreviations are as follows: H = Holocene, M = Magdalenian, G = Gravettian, A/G = A/G transition, A = Aurignacian. For the rock types: RS = Red sediment, DL = Degraded Limestone. Table I: Hohle Fels ochre textures sorted by archaeological horizon (AH) and time period. Abbreviations are as follows: H = Holocene, M = Magdalenian, G = Gravettian, A/G = A/G transition, A = Aurignacian. For the textures: Med. Sand = Medium grained sand. Table J: Hohle Fels ochre colours sorted by archaeological horizon (AH) and time period. Abbreviations are as follows: H = Holocene, M = Magdalenian, G = Gravettian, A/G = A/G transition, A = Aurignacian. For the colours: BR = Brick Red, DP = Dark Purple, DR = Dark Red, LP = Light Purple, LR = Light Red. (PDF) [file pone.0209874.s002.pdf]

Ochre and pigment use at Hohle Fels cave: Results of the first systematic review of ochre and ochre-related artefacts from the Upper Palaeolithic in Germany

Elizabeth C. Velliky*, Martin Porr, Nicholas J. Conard

* Corresponding author

E-mail: elizabeth.velliky@research.uwa.edu.au

S1 Tables A-J. Artefact numbers, contextual details, and statistical tests for corresponding figures.

Table A: Figure 3 (Hohle Fels Aurignacian) artefact descriptions. 2

Table B: Figure 4 (Hohle Fels Gravettian) artefact descriptions. 3

Table C: Figure 5 (Hohle Fels Magdalenian) artefact descriptions. 4

Table D: Figure 10 (Hohle Fels artefacts with residues) artefact descriptions. 5

Table E: Means and standard deviations for L*A*B* values per time period. 6

Table F: One-way ANOVA results for measured L* values for each time period. 7

Table G: Tukey-Kramer post-hoc test data for L* (lightness) colour values on Hohle Fels colour streaks per time period. 8

Table H: Hohle Fels ochre rock type sorted by archaeological horizon (AH) and time period. Abbreviations are as follows: H = Holocene, M = Magdalenian, G = Gravettian, A/G = A/G transition, A = Aurignacian. For the rock types: RS = Red sediment, DL = Degraded Limestone. 9

Table I: Hohle Fels ochre textures sorted by archaeological horizon (AH) and time period. Abbreviations are as follows: H = Holocene, M = Magdalenian, G = Gravettian, A/G = A/G transition, A = Aurignacian. For the textures: Med. Sand = Medium grained sand. 10

Table J: Hohle Fels ochre colours sorted by archaeological horizon (AH) and time period. Abbreviations are as follows: H = Holocene, M = Magdalenian, G = Gravettian, A/G = A/G transition, A = Aurignacian. For the colours: BR = Brick Red, DP = Dark Purple, DR = Dark Red, LP = Light Purple, LR = Light Red. 11

Table A: Figure 3 (*Hohle Fels* Aurignacian) artefact descriptions.

Aurignacian	Year	Quad	AH	Find #
1	2013	54	IV	557
2	2002	58	IId-IV	2099.2
3	2002	58	IId-IV	2099.1
4	2002	57	IId	3149
5	2016	31	Vaa	2095
6	2013	27	Va	1271
7	2016	32	Vaa	1635
8	2012	29	Vb	1966
9	2014	28	Vab	1856
10	2006	86	IV	1122.1
11	2003	59	V-VII	2365
12	2004	87	IIIa.1	1205
13	2013	54	IIIa	338
14	1996	79	IId	2224
15	2016	32	Vaa	1635.2
16	2007	29	Va	1838
17	2000	79	IIe	2555
18	1995	79	IId	2083
19	2015	31	IIIa	984.2
20	2015	32	IV	215
21	2006	86	IV	1122.2
22	2015	32	Va	1079
23	2015	31	IIIa	984.1
24	2004	75	IV	631
25	2016	32	Vaa	1635.1
26	2005	86	IIIa	975
27	2004	97	IIIa.1	1054
28	2006	99	IId-IV	2087
29	2013	54	IIIa	363.1
30	2005	98	IV.6	1702
31	2004	66	IIIa-V	4347

Table B: Figure 4 (Hohle Fels Gravettian) artefact descriptions.

Gravettian	Year	Quad	AH	Find #
1	1998	56	IIb	404
2	1998	65	IIb-IVb	205.1
3	2017	111	IIb	621
4	1995	58	IIb	571
5	2002	56	IIcf	3502.1
6	1998	65	IIb-IV	205.3
7	1977	12	IIbf	54
8	2004	55	IIc	1256
9	1977	12	IIbf	54.2
10	2011	136	IIb	328
11	1990	79	IIb	887
12	1977	12	IIbf	54.3
13	2012	46	IIb	251
14	2007	30	IIdb	754
15	1995	56	IIb	245
16	1995	56	IIb	245.2
17	1998	56	IIb	358.1
18	1995	57	IIb	444
19	1995	57	IIb	443
20	1995	67	IIb	350.1
21	1998	65	IIb-IV	205.2
22	1990	79	IIb	852
23	1995	56	IIb	278
24	1998	56	IIb	358.2
25	2010	110	IIb	1166
26	1995	58	IIb	572
27	1977	12	IIbf	54.1
28	1988	59	IIb	867
29	1995	67	IIb	350.2
30	1998	56	IIb	454
31	1990	79	IIb	865
32	1998	65	IIb-IV	205.4

Table C: Figure 5 (Hohle Fels Magdalenian) artefact descriptions.

Magdalenian	Year	Quad	AH	Find #
1	2011	111	IIad	357
2	2011	112	IIaf	475
3	2010	112	IIaf	547
4	95	68	I	826
5	1993	67	I	147
6	1991	68	IIa	268
7	2001	89	I	129.1
8	1998	55	I	276
9	2002	99	0I	132
10	1978	24	IIa	380
11	2001	89	I	129
12	2009	102	IIaf	693
13	1992	67	I	52
14	2010	110	IIad	1104
15	2010	110	IIad	971
16	1978	59	Ia	214
17	2001	89	I	179
18	1979	24	Ic	531
19	94	67	IIa	208

Table D: Figure 10 (Hohle Fels artefacts with residues) artefact descriptions.

			Residues			
	Period	Year	Quad	AH	Find #	Material
1	Aurignacian	1996	59	IV	1784	Reindeer rib
2	Magdalenian	1988	79	IIa	3592	
3	Aurignacian	2004	97	IIIa	1083	Jurahornstein
4	Aurignacian	2003	76	IV	1510.1	Ivory bead
5	Aurignacian	2009	25	Vab	1340.2	Ivory bead
6	Aurignacian	2009	25	Vab	1310.2	Ivory bead
7	Aurignacian	2014	25	Vab-VII	1354.2	Ivory bead
8	Aurignacian	2013	27	Va	1011	Ivory bead
9	A/G trans	2000	79	IIe	2555.108	
10	Magdalenian	1988	79	IIa	314.1	Rabbit tooth
11	Magdalenian	1988	79	IIa	314.2	Fox tooth
12	Magdalenian	1988	49	Ib	158	Shell
13	Magdalenian	1977	11	IIa	82	Shell
14	Gravettian	1995	67	IIb	367	
15	Magdalenian	2001	59	0-IIIb	1842	Limestone
16	Aurignacian	2009	99	IV.6	2262	Jurahornstein
17	A/G trans	2000	79	IIe	2555.109	
18	Gravettian	1988	69	IIb	232	Shell
19	Gravettian	2006	100	IIb	598	Shell
20	Gravettian	1994	67	IIb	222	Shell
21	Magdalenian	2015	110	0/I-IId	1314	Mollusc fossil
22	Aurignacian	2005	98	IIIawf	1254	

Table E: Means and standard deviations for L*A*B* values per time period.

L (lightness)*

Period	n	Mean	SD	SE	Lower 95%	Upper 95%
A/G Trans	25	62.8	20.78	4.16	54.22	71.38
Aurignacian	286	70.52	17.86	1.06	68.44	72.6
Gravettian	217	55.72	16.46	1.12	53.52	57.93
Holocene	13	52.92	12.22	3.39	45.54	60.31
Magdalenian	120	53.03	14.07	1.28	50.48	55.57

A (green-red)*

Period	n	Mean	SD	SE	Lower 95%	Upper 95%
A/G Trans	25	11.56	10.53	2.11	7.21	15.91
Aurignacian	286	11.04	10.89	0.64	9.77	12.30
Gravettian	217	16.38	9.25	0.63	15.14	17.62
Holocene	13	18.92	6.73	1.87	14.86	22.99
Magdalenian	120	19.12	7.53	0.69	17.76	20.48

B (blue-yellow)*

Period	n	Mean	SD	SE	Lower 95%	Upper 95%
A/G Trans	25	32.92	12.65	2.53	27.7	38.14
Aurignacian	286	36.81	10.39	0.62	35.6	38.02
Gravettian	217	30.13	12.29	0.83	28.5	31.78
Holocene	13	30	5.57	1.54	26.64	33.37
Magdalenian	120	32.28	9.16	0.84	30.62	33.93

Table F: One-way ANOVA results for measured L* values for each time period.

Analysis of Variance

Groups	df	Sum of Squares	Mean Square	F	Prob > F
Between	4	40034.90	10008.7	35.4745	*<.0001**
Within	656	185082.67	282.1		
Total	660	225117.57			

Means for Oneway Anova

Period	n	Mean	SE	Lower 95%	Upper 95%
A/G Trans	25	62.80	3.36	56.20	69.40
Aurignacian	286	70.52	0.99	68.57	72.47
Gravettian	217	55.72	1.14	53.49	57.96
Holocene	13	52.92	4.66	43.78	62.07
Magdalenian	120	53.03	1.53	50.01	56.04

Table G: Tukey-Kramer post-hoc test data for L* (lightness) colour values on Hohle Fels colour streaks per time period.

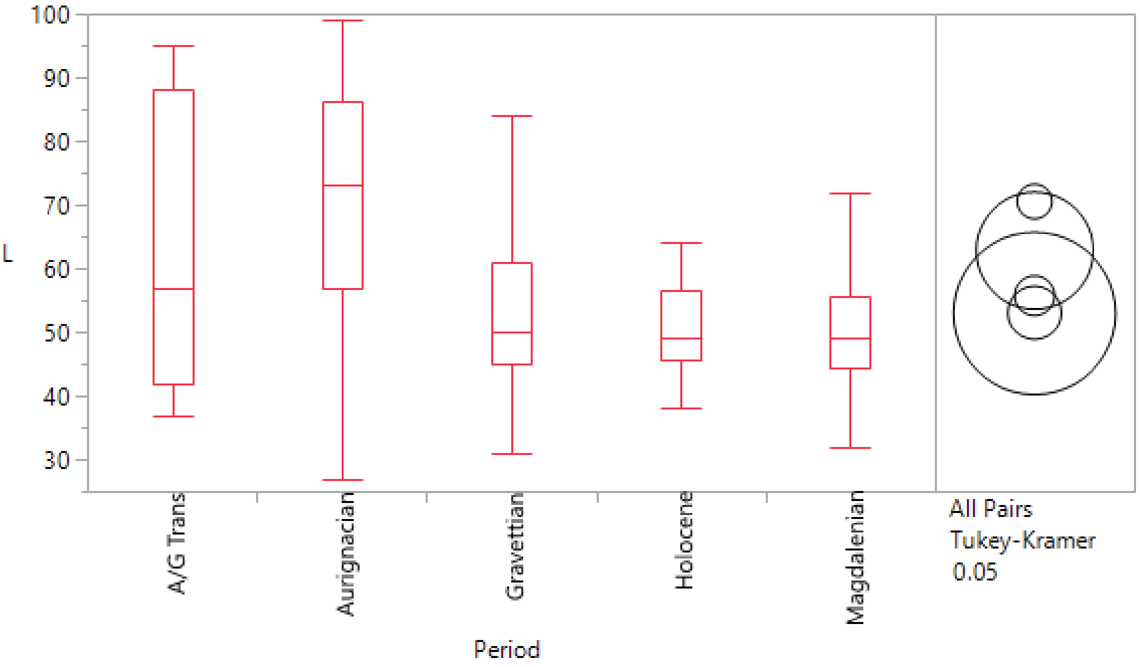

Ordered Differences Report from Tukey-Kramer's test on each pair

q = 2.73540, α = 0.05, starred (*) and italicized values are statistically significantly different pairs.

Group comparisons		Difference	SE	Lower CL	Upper CL	p-Value
Aurignacian	Holocene	17.59441	4.763343	4.5647	30.62407	*0.0022**
Aurignacian	Magdalenian	17.49248	1.826923	12.4951	22.48986	*<.0001**
Aurignacian	Gravettian	14.79398	1.512175	10.6576	18.93039	*<.0001**
A/G Trans	Holocene	9.87692	5.743559	-5.8340	25.58788	0.4225
A/G Trans	Magdalenian	9.77500	3.692788	-0.3263	19.87627	0.0633
Aurignacian	A/G Trans	7.71748	3.503145	-1.8650	17.30000	0.1798
A/G Trans	Gravettian	7.07650	3.547634	-2.6277	16.78071	0.2697
Gravettian	Holocene	2.80043	4.796156	-10.3190	15.91985	0.9774
Gravettian	Magdalenian	2.69850	1.910845	-2.5284	7.92543	0.6200
Magdalenian	Holocene	0.10192	4.904497	-13.3139	13.51770	1.0000

Table H: Hohle Fels ochre rock type sorted by archaeological horizon (AH) and time period. Abbreviations are as follows: H = Holocene, M = Magdalenian, G = Gravettian, A/G = A/G transition, A = Aurignacian. For the rock types: RS = Red sediment, DL = Degraded Limestone.

AH	Period	Total	Volume	Clay	Hematite	Iron Oxide	RS	Sandstone	Siltstone	DL	Specularite
0-0/I	H	21	9814.18	1	14	3		2	1		
I	M	93	65669.22	5	70	6	2	8	2		0
IIa	M	71	112625.5	2	42	13		12	2		
IIb	G	227	208972.9	1	172	31	2	21			
IIc	G	43	29283.49	2	11	15	2	12	1		
IIcf	G	8	14820.5		2		1	5			
IId	A/G	29	111990	1	7	12	1	7		1	
IIe	A/G	6	14644.24		2	4					
IIIa	A	52	256249.6	1	27	5	1	15		2	1
IIIb	A	3	793.61		2	1					
IV	A	67	19292.87	1	11	16	15	22	1		1
Va	A	241	59783.42	3	51	42	17	124	3		1
Vb	A	8	2756.56		2	2	1	3			
**Total**		**869**	**877412.6**	**17**	**413**	**150**	**42**	**231**	**10**	**3**	**3**

Table I: Hohle Fels ochre textures sorted by archaeological horizon (AH) and time period. Abbreviations are as follows: H = Holocene, M = Magdalenian, G = Gravettian, A/G = A/G transition, A = Aurignacian. For the textures: Med. Sand = Medium grained sand.

AH	Period	Total	Volume	Clayey	Clayey/Silty	Silty	Silty/Sandy	Very fine sand	Fine sand	Med. sand
0-0/I	H	21	9814.18	2	1	5	7	3	3	
I	M	93	65669.22	13	17	15	31	6	10	1
IIa	M	71	112625.5	10	8	10	19	11	11	2
IIb	G	227	208972.9	26	69	18	69	9	33	3
IIc	G	43	29283.49	5	6	5	6	13	6	2
IIcf	G	8	14820.5					2	6	
IId	A/G	29	111990	3	1	6	6	4	8	1
IIe	A/G	6	14644.24			4	2			
IIIa	A	52	256249.6	1		25	3	5	15	3
IIIb	A	3	793.61			2	1			
IV	A	67	19292.87	4	4	5	8	9	31	6
Va	A	241	59783.42	31	15	12	18	19	134	12
Vb	A	8	2756.56	1		1	1	1	3	1
**Total**		**869**	**877412.6**	**96**	**121**	**108**	**171**	**82**	**260**	**31**

Table J: Hohle Fels ochre colours sorted by archaeological horizon (AH) and time period. Abbreviations are as follows: H = Holocene, M = Magdalenian, G = Gravettian, A/G = A/G transition, A = Aurignacian. For the colours: BR = Brick Red, DP = Dark Purple, DR = Dark Red, LP = Light Purple, LR = Light Red.

**AH**	**Period**	**Total**	**Volume**	**Black**	**Brown**	**BR**	**DP**	**DR**	**LP**	**LR**	**Orange**	**Pink**	**Purple**	**Red**	**Rust**	**Yellow**
0-0/I	H	21	9814.18				5	5	1	2		1	7			
I	M	93	65669.22				34	8	4	5	1	1	31	9		
IIa	M	71	112625.5		1		24	6	1	6		2	17	13	1	
IIb	G	227	208972.9			2	52	5	6	14	1	6	117	19	5	
IIc	G	43	29283.49	1	1	5	9	3		9	1	1	5	7	1	
IIcf	G	8	14820.5					1		5			2			
IId	A/G	29	111990			1	4	5		3	3		4	4	5	
IIe	A/G	6	14644.24				3		1				1			1
IIIa	A	52	256249.6	1		10	28	2		2				5	3	1
IIIb	A	3	793.61	1	1		1									
IV	A	67	19292.87		6	2	8	7		12		2	5	25		
Va	A	241	59783.42	2	1	28	47	37	1	47	1	2	13	58	3	1
Vb	A	8	2756.56				2	2				1		3		
**Total**		**869**	**877412.6**	**5**	**10**	**48**	**217**	**81**	**14**	**105**	**7**	**16**	**202**	**143**	**18**	**3**
